# Programmable Lipid Functionalization of Nucleic Acid Nanoparticles Modulates Liver Cell-Type Targeting

**DOI:** 10.1021/acsami.5c24581

**Published:** 2026-03-25

**Authors:** Hyun Min Kim, Marjan Omer, Grant A. Knappe, Patrick McMullen, Duy An Le, Ashwin Pasupathy, Daniel G. Anderson, Mark Bathe

**Affiliations:** † Department of Biological Engineering, 2167 Massachusetts Institute of Technology, Cambridge, Massachusetts 02139, United States; ‡ Department of Biomedicine, Aarhus University, 8000 Aarhus C, Denmark; § Department of Chemical Engineering, Massachusetts Institute of Technology, Cambridge, Massachusetts 02139, United States; ∥ David H Koch Institute for Integrative Cancer Research, Massachusetts Institute of Technology, Cambridge, Massachusetts 02139, United States; ⊥ Department of Anesthesiology, Boston Children’s Hospital, Boston, Massachusetts 02115, United States; # Harvard and MIT Division of Health Science and Technology, Massachusetts Institute of Technology, Cambridge, Massachusetts 02139, United States; ∇ Institute for Medical Engineering and Science, Massachusetts Institute of Technology, Cambridge, Massachusetts 02139, United States; ○ Broad Institute of MIT and Harvard, Cambridge, Massachusetts 02142, United States; ◆ Harvard Medical School Initiative for RNA Medicine, Harvard Medical School, Boston, Massachusetts 02115, United States

**Keywords:** drug delivery, nucleic acid therapeutics, nanoparticles, DNA origami, multivalency, biomolecular corona

## Abstract

Nucleic acid nanoparticles (NANPs) fabricated by using DNA origami are an emerging delivery vector for nucleic acid therapeutics. Despite their advantages over other nanomaterials that include controlled spatial presentation of targeting ligands such as lipids and sugars, understanding their cell targeting and uptake mechanisms remains limited. Here, we investigated NANP cellular targeting, uptake, and delivery of small interfering RNAs (siRNAs) to liver and neuronal cell models *in vitro*. Using a rational design approach, we targeted NANPs to two clinically validated receptors, the asialoglycoprotein receptor (ASGPR) and the low-density lipoprotein receptor (LDLR), respectively, using GalNAc and lipidation. We systematically evaluated how the ligand valency, interligand spacing, linker length, and ligand chemistry affected NANP association with on- and off-target liver cell types, revealing the relative roles of the biomolecular corona, receptor engagement, and endocytosis in these targeting strategies. We found that lipidation enhanced NANP uptake into HepG2 cells, a model cell line for hepatocytes, by promoting apolipoprotein recruitment, LDLR engagement, and clathrin-mediated endocytosis and also increased association with nonparenchymal cells. HepG2 uptake was further improved by conjugating NANPs to lipids with higher valency provided that lipids were adequately displayed away from the surface of NANP edges with more lipophilic lipids yielding greater cell association. We then benchmarked the potential for NANPs to deliver siRNAs to HepG2 cells in comparison with lipid nanoparticle and conjugate technologies and explored lipid functionalization as a strategy for nonhepatic NANP targeting to model neuronal cells. Overall, this study advances the foundational understanding of how clinically relevant targeting ligands mediate NANP interactions with both on- and off-target liver cell types *in vitro*, offering insights into potential design criteria for nucleic acid therapeutic delivery.

## Graphical abstract

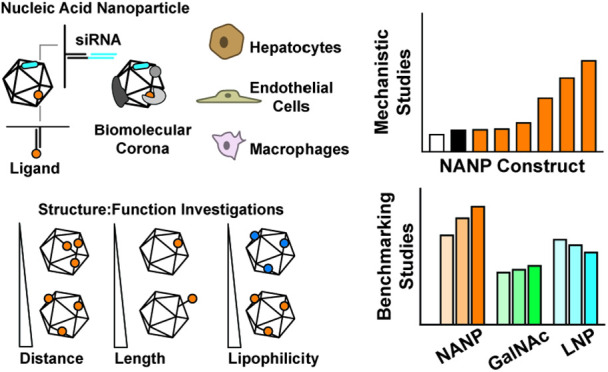

## Introduction

Significant advances over decades in the design, chemistry, and formulation of nucleic acid therapeutics such as small interfering RNAs (siRNAs) have led to the FDA approval of over 20 medicines.^1,2^ A major driver of these successes has been the development of nonviral delivery technologies, in particular lipid nanoparticles (LNPs) and *N*-acetylgalactosamine (GalNAc) conjugate systems, which have enabled clinical siRNA delivery to the liver. Despite this progress, targeted delivery of siRNA to nonhepatic cell types remains a major barrier to their broader clinical translation, due in part to the high molecular weight, negative charge, and rapid renal clearance of siRNAs that limit extrahepatic targeting and therapeutic efficacy. In addition, strategies to realize safe and effective nonhepatic targeted delivery of high-molecular-weight and complex nucleic acid therapeutics such as mRNA, gene-length ssDNA templates, and ribonucleoprotein complexes remain limited. Thus, new delivery technologies are needed.

LNPs encapsulate therapeutic cargo to protect against degradation, target hepatocytes through cell-surface receptors, and release cargo into the cytosol through endosomal barrier modulation.^3^ To target hepatocytes, LNPs recruit apolipoproteins such as apolipoprotein E (ApoE) to mimic very low, low, and high density lipoproteins (VLDLs, LDLs, and HDLs) and subsequently engage low density lipoprotein receptors (LDLR) and scavenger receptor class B type I (SR-B1) to initiate receptor-mediated endocytosis.^2,4^ Despite their clinical successes, LNPs face several limitations, including dose-limiting toxicity,^5^ inability to be administered subcutaneously, poorly characterized immunogenicity,^6^ limited endosomal escape efficiency,^7^ and limited extrahepatic targeting capabilities.^3^ Other nonviral carrier strategies under development include polymeric nanoparticles,^8^ exosomes,^9^ and protein-based virus-like particles (P-VLPs).^10^ These technologies similarly encapsulate cargo, promote on-target accumulation via surface chemistry modulation, and attempt to achieve endosomal escape, with preclinical and clinical evidence supporting their utility. Despite their respective advantages, all of these platforms continue to face key challenges including consistent manufacturing, uncontrollable immunogenicity, dose-limiting toxicity, and limited cell-specific targeting capabilities.

Contemporaneously, carrier-free strategies were developed as oligonucleotide chemical modifications emerged to enhance siRNA stability *in vivo*,^1^ most notably the trivalent GalNAc (TriGalNAc) conjugate systems that bind to the asialoglycoprotein receptor (ASGPR) specifically expressed on hepatocytes.^1^ These have enabled simpler manufacturing, highly specific targeting, favorable safety profiles, and subcutaneous administration with improved durability due to an endosomal depot effect that leads to prolonged exposure and sustained gene silencing.^11^ The success of the GalNAc delivery system has motivated the development of numerous other conjugate-based technologies. Lipid–siRNA conjugates are another carrier-free strategy explored extensively in preclinical and clinical studies toward enabling extrahepatic siRNA delivery. Lipid–siRNAs were the first conjugates to be used for systemic siRNA delivery,^12^ and advanced chemical engineering of lipid–siRNA conjugates has now demonstrated enhanced siRNA accumulation and functional gene silencing in extrahepatic tissues including the brain, lung, heart, muscle, and fat, establishing lipid conjugation as a promising strategy for broadening the therapeutic reach of RNA interference (RNAi).^13,14^ Antibody–siRNA conjugates and similar protein-based systems are also emerging as additional clinical delivery systems that enable cell-type-specific uptake and codelivery of gene-silencing and immunostimulatory payloads.^15,16^ Aptamer–siRNA conjugates offer a complementary, entirely nucleic acid-based alternative for cell- and receptor-specific delivery, with several preclinical studies demonstrating selective uptake and gene silencing.^17^ However, these conjugate systems still face notable challenges in their abilities to deliver high-molecular-weight nucleic acids like plasmid DNA, single-stranded DNA, and mRNA and minimal control over endosomal fate.^18^

A distinct, early-stage technology with potential for enabling oligonucleotide delivery is nucleic acid nanoparticles (NANPs). NANPs are self-assembled nanoparticles comprised of nucleic acids that are designed and fabricated using the scaffolded DNA origami method.^19,20^ This programmable process folds a long single-stranded DNA (ssDNA) “scaffold” into user-defined nanoscale objects through complementary base pairing with short oligonucleotide “staples”. In principle, NANPs may offer several advantages for delivering nucleic acid therapeutics.^21,22^ First, because NANPs are composed of nucleic acids themselves, nucleic acid therapeutics can be coformulated with the delivery vector in a facile manner, including stoichiometrically controlled loading of therapeutics against multiple targets. Second, NANP size, shape, and other physicochemical properties such as charge and hydrophobicity are controllable through strategies including oligolysine-PEGylation^23^ and chemical modifications,^1^ potentially enabling the design of delivery vectors that simultaneously optimize on-target and off-target trafficking through passive and active distribution strategies.^24–26^ Third, their programmable architecture allows for multivalent display of ligands at precise positions,^20,21^ a feature that can enhance avidity-driven interactions with cell-surface receptors.^24,27,28^ While multivalency is employed by other platforms such as GalNAc conjugates^1^ and P-VLPs,^10^ DNA origami uniquely enables spatially structured control of multivalency with nanometer precision, which is a material property with significant potential that remains vastly under-explored both *in vitro* and *in vivo*. Finally, NANPs are biodegradable,^29^ lack IgG-mediated antibody responses against the vector,^28^ and have well-defined immunostimulatory properties that can be modulated through chemical engineering.^30–33^

Accordingly, NANPs have been deployed extensively in prior work to deliver siRNAs and ASOs,^34–37^ particularly in the context of cancer and immunotherapy. Notwithstanding, despite their preceding positive attributes, NANPs also face several key limitations as delivery vectors, including their susceptibility to degradation by endogenous nucleases^29^ and their rapid clearance via scavenger receptors of the mononuclear phagocyte system (MPS) due to their high negative charge.^38–40^ Furthermore, cytosolic double-stranded DNA (dsDNA) can activate the cGAS-STING pathway in a molecular-weight-dependent, sequence-independent manner that may lead to immunotoxicity,^41^ and it remains unclear if and in what contexts NANPs might lead to anti-DNA autoimmunity.^28,32^ Finally, NANPs are typically not benchmarked against state-of-the-art delivery technologies, creating a knowledge gap around their comparative advantages as a technology class. Thus, mechanistic *in vitro* cell-based studies elucidating the mechanisms of NANP cell targeting, uptake, and gene silencing, particularly in the context of lipidation strategies, remain needed.

Systemically administered delivery systems must navigate biological barriers such as blood or lymph proteins and the MPS, and they must traverse the fenestrated sinusoidal endothelium to access the liver parenchyma. There, they must further avoid nonspecific sequestration by liver sinusoidal endothelial cells (LSECs) and Kupffer cells (liver-resident macrophages), in order to subsequently deliver siRNAs to hepatocytes.^39,40^ LNPs are typically administered intravenously and achieve hepatocyte delivery through PEGylation to evade MPS sequestration, controlled particle sizes under 100 nm to facilitate parenchymal access, and ApoE adsorption to enable LDLR-mediated endocytosis.^3,4^ GalNAc conjugates enter hepatocytes via ASGPR-mediated endocytosis, as their small size and defined chemistry enable them to largely bypass protein interactions and MPS uptake, and their subcutaneous mode of administration promotes lymphatic transport. In contrast to these well-understood technologies, how NANPs interface with these same biological barriers remains poorly understood.

Like other nanomaterials, the liver is the primary site of accumulation for NANPs following intravenous administration,^32,38,42^ possibly due to rapid accumulation within Kupffer cells. NANP–macrophage interactions have been extensively studied, with general observations that NANPs strongly associate with macrophages due to scavenger receptors.^43,44^ In addition, NANPs face degradation *in vivo* as the ubiquitous endonuclease DNase I is also highly expressed in the liver where NANPs accumulate.^29^ PEGylation, routinely implemented in other nanomaterial systems,^40^ is often employed through oligolysine–PEG copolymer coating of NANPs that stabilize assemblies through electrostatic interactions, impart stability against nuclease degradation, and decrease association with macrophages,^23,45,46^ presumably through steric blocking of receptors and modulating the biomolecular corona.^45,47^ These strategies are routinely implemented *in vivo* for extending circulation lifetimes and enabling cell-specific targeting,^23,24,27^ although a recent whole-body murine single-cell imaging study revealed NANPs with this PEGylation approach still primarily accumulated in the liver after 20 min, suggesting additional development is required for detargeting the liver.^38^ Researchers have alternatively turned to programming the biomolecular corona on NANPs to achieve desired distributions, with a recent proof-of-concept in recruiting lipoproteins to target liver cells.^37^ Despite these advances, our understanding of how the NANP structure modulates biomolecular corona formation and cell targeting remains limited. In addition, previous studies often do not benchmark NANPs against clinically established oligonucleotide delivery platforms such as LNPs and GalNAc conjugates, limiting our comparative understanding of how NANPs perform in terms of targeting specificity and potency. To address these knowledge gaps, additional mechanistic investigations of NANP–liver cell dynamics are warranted.

In order to reveal mechanistic aspects of cell targeting and uptake of NANPs mediated by lipidation and GalNAc, we used *in vitro* cell models to investigate several key NANP properties known to modulate cellular interactions, including the lipid type, valency, spatial organization, and linker distance, investigating how to optimize NANP association with HepG2 cells, a liver cancer cell line that is widely used as a human hepatocyte model. Our study provided insights into the relative roles of protein recruitment within the biomolecular corona, receptor engagement, and endocytosis pathways that enabled NANP internalization into HepG2 cells. We additionally explored off-target cell association with nonparenchymal liver cell types using SK-HEP-1 and J774A.1 cell lines as models for LSECs and Kupffer cells, respectively, and benchmarked NANPs to state-of-the-art LNP and GalNAc delivery systems. We then evaluated the transferability of the knowledge gained from the preceding cell lines to a nonhepatic, model neuronal cell line, given the ongoing need for new oligonucleotide delivery strategies for central nervous system (CNS) applications. Taken together, our work helps to advance our understanding of NANPs as a platform for nucleic acid therapeutic delivery.

## Results and Discussion

### Fabrication and Characterization of Carbohydrate- and Lipid-Modified NANPs

To systematically evaluate the targeting capabilities of NANPs, we investigated hepatocyte targeting via two clinically validated strategies—active ASGPR targeting and passive LDLR targeting—in model liver cell lines. We designed and fabricated a library of particles based on an icosahedral NANP composed of 52 base pair (bp) long, double crossover (DX) edges (termed ICO) that we have extensively investigated previously.^28,48^ ICO is approximately 35 nm in diameter, within the theoretical range for minimizing clearance by both the renal and MPS systems.^39,40^ Select staples were modified to include outward-facing 3′ 20-nt ssDNA extensions to enable ligand and cargo loading. Ligand-functionalized oligonucleotides and siRNAs (gift from Novo Nordisk Global Nucleic Acid Therapies) were synthesized with complementary nucleic acid sequences to enable formulation into the NANPs at specific copy numbers and positions on the nanostructure (Figures 1a and S1). TriGalNAc and cholesterol were chosen as ligands to target the ASGPR and LDLR pathways, respectively.^37^ Select staples were labeled with Alexa Fluor 647 (AF647) preassembly via click chemistry to facilitate detection of cellular association and internalization assays.

**Figure 1:**
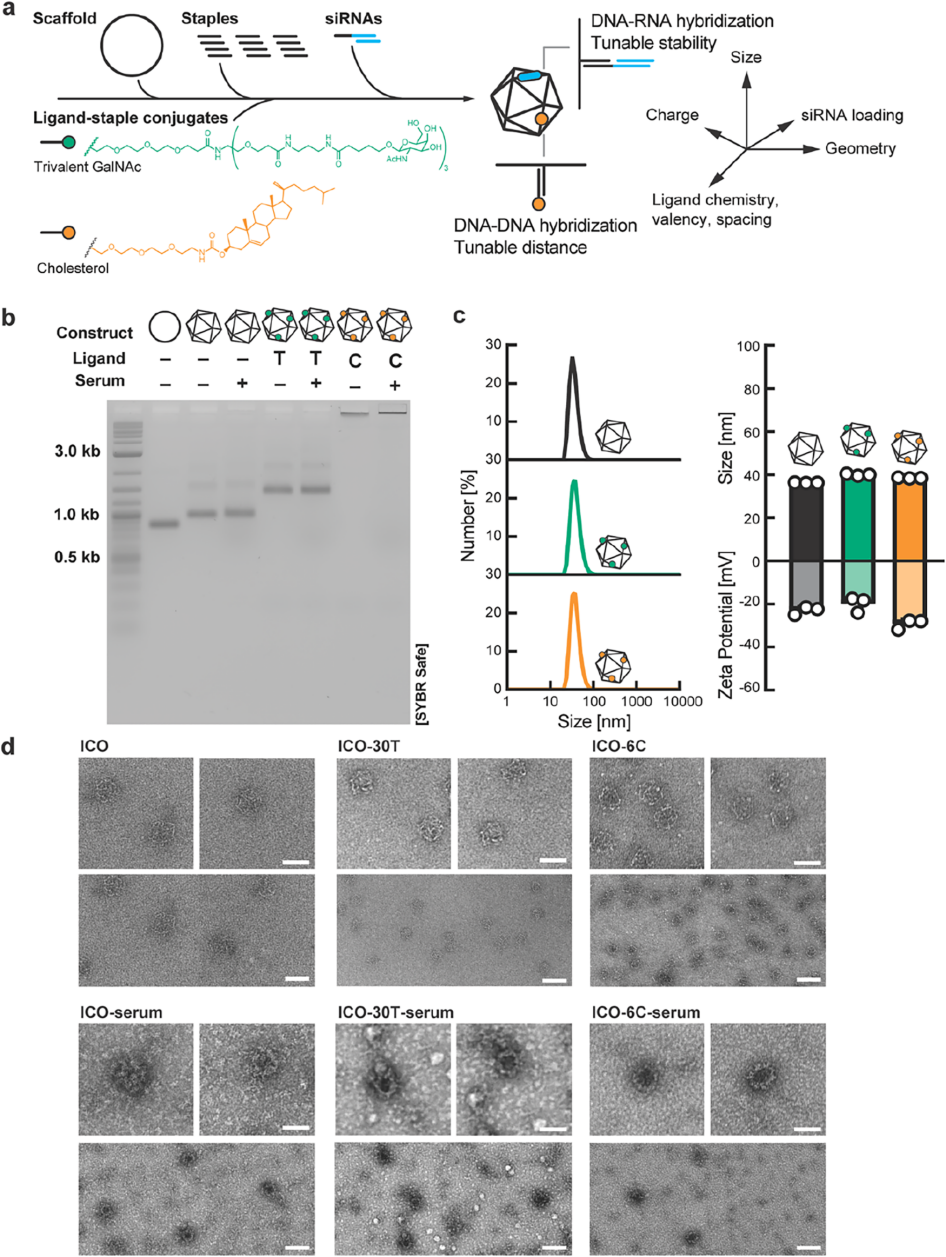
Fabrication and characterization of liver-targeted icosahedral NANPs. (a) Schematic of NANP manufacturing and design space. (b) AGE analysis demonstrates reduced mobility for assembled NANPs, as well as for NANPs displaying ligands, specifically 30 copies of TriGalNAc (T) or 6 copies of cholesterol (C), with and without preincubation in 55% human serum to simulate biomolecular corona formation. (c) Hydrodynamic diameters and ζ-potentials of unmodified and ligand-functionalized ICOs measured by DLS. (d) TEM micrographs of bare and ligand-conjugated ICOs, with and without serum incubation, confirm the structural integrity and morphology following ligand functionalization and biomolecular corona formation. Scale bars represent 50 nm (top) and 100 nm (bottom).

Ligand-functionalized NANPs were assembled through thermal annealing of the DNA scaffold and complementary staple sets including modified staples for ligand and dye incorporation (Tables S1–S3). Agarose gel electrophoresis (AGE) confirmed NANP self-assembly with distinct bands corresponding to the scaffold, ICO, TriGalNAc-modified ICO (ICO-*n*T), and cholesterol-modified ICO (ICO-*n*C) (Figure 1b). ICO-6C minimally migrated, likely due to the incorporation of hydrophobic cholesterol molecules, which generally impedes NANP mobility in AGE, consistent with previous reports.^49,50^ Dynamic light scattering (DLS) confirmed monodisperse assemblies for ICO, ICO-6T, and ICO-6C, with hydrodynamic diameters consistent with theoretical dimensions (Figure 1c). ζ-Potential measurements confirmed that these NANPs are negatively charged with ζ-potentials between −20 and −30 mV. The incorporation of ligands minimally altered the particle size, polydispersity, and charge. Transmission electron microscopy (TEM) additionally confirmed the size, morphology, and structural integrity of the NANPs. When the NANPs were incubated in 55% human serum, to mimic *in vivo* biomolecular corona formation,^4^ TEM imaging revealed monodisperse NANPs surrounded with additional electron density, which we attribute to adsorbed biomacromolecules (Figure 1d).

### Modeling the Association of Ligand-Modified NANPs with Hepatocytes, LSECs, and Kupffer Cells *In Vitro*

We evaluated the *in vitro* cellular association of TriGalNAc- and cholesterol-modified ICOs with the three major liver cell types using human HepG2 cells to model hepatocytes,^10,11,51^ human SK-HEP-1 cells to model LSECs,^10^ and murine J774A.1 cells to model Kupffer cells (Figures 2a–c, S2, and S3).^52^ To validate the relevance of these models for our study, we confirmed the anticipated expression profiles of ASGPR, LDLR, SR-B1, and SR-A with immunostaining (Figure S4) following previously published protocols for these systems.^53^ For cellular association studies, cells were seeded onto 48-well plates and incubated with NANPs for 1 h; cell association was subsequently assayed using flow cytometry. Cell association includes both surface binding and particle internalization unless otherwise specified. NANPs were preincubated in 55% human serum before administration onto cells to better model the *in vivo* environment.^4^

**Figure 2:**
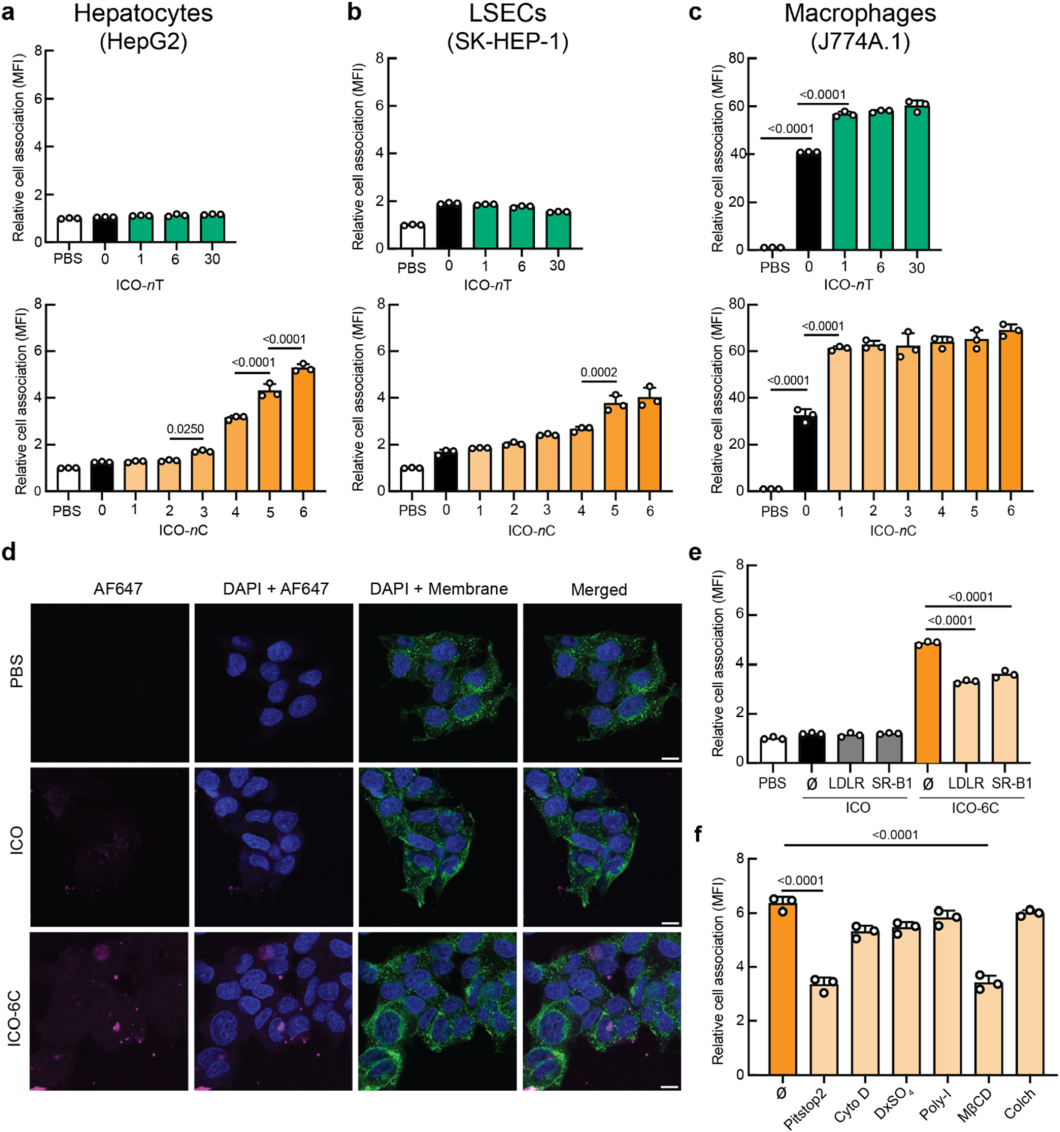
Lipid functionalization drives cellular internalization of NANPs into liver cell types via receptor-mediated endocytosis. (a–c) Relative association of AF647-labeled NANPs functionalized with increasing numbers of TriGalNAc (top row) or cholesterol (bottom row) following 1 h of incubation at 50 nM with HepG2, SK-HEP-1, and J774A.1 cells. Cell association was quantified by flow cytometry as mean fluorescence intensity (MFI) and the data were normalized to the PBS control group. (d) Confocal microscopy (63×) of AF647-labeled ICOs (magenta) in HepG2 cells after 24 h. Membrane stained with Wheat Germ Agglutinin AF488 (green) and nuclei with DAPI (blue). Scale bars represent 10 μm. (e) HepG2 association of ICO and ICO-6C following preincubation with monoclonal antibodies against LDLR and SR-B1. (f) Effects of clathrin inhibition (Pitstop 2), actin-dependent macropinocytosis inhibition (cytochalasin D), SR-A inhibition (dextran sulfate), SR-A and SR-B inhibition (poly-I), lipid raft disruption, and caveolin inhibition through membrane cholesterol depletion (MβCD), and microtubule disruption (colchicine) on ICO-6C association with HepG2 cells. For panels (a–c) and (e, f), error bars indicate mean ± SD, *n* = 3 biological replicates. An ordinary one-way ANOVA with Tukey’s multiple comparisons test (α = 0.05) was conducted; *p*-values indicated. Ø: no inhibitor; Cyto D: cytochalasin D; DxSO_4_: dextran sulfate; poly-I: polyinosinic acid; Colch: colchicine.

We hypothesized that the multivalent display of TriGalNAc and cholesterol would enhance NANP association to HepG2 cells due to receptor-mediated endocytosis through ASGPR and LDLR, respectively. This hypothesis was supported by multivalent effects observed in lipid nanoparticle,^2^ nucleic acid,^51^ and protein nanoparticle systems.^10^ To explore multivalency effects, we fabricated ICO-1T, -6T, -30T, and ICO-1C, -2C, -3C, -4C, -5C, -6C (Figure S1a,b), based on previous studies identifying the valency ranges relevant for each ligand system,^10,37^ and evaluated their cellular association. We observed that ICO-1T, -6T, and -30T did not associate with HepG2 cells, while cholesterol-functionalized NANPs did associate with HepG2 cells in a valency-dependent manner, with up to a 6-fold increase in mean fluorescence intensity (MFI) over the PBS-treated group (Figure 2a).

To confirm the bioactivity of TriGalNAc after formulation with NANPs, we assayed binding of recombinant, soluble ASGPR to ICO-30T in a gel mobility shift assay. ICO-30T had reduced mobility when ASGPR was present, confirming TriGalNAc was present and bioactive in the formulations (Figure S5a). To ensure the failure of the TriGalNAc targeting strategy was not an assay artifact, we conducted a similar cell association study with TriGalNAc-modified 20mer dsDNA duplex, a previously published, multivalent Holliday junction,^51^ and a previously published, larger multivalent tetrahedron^36^ (Figure S5b,c and Tables S5, S6). We observed a valency-dependent association of cells with these alternative nucleic acid systems. Thus, we do not attribute the lack of cell association of TriGalNAc-targeted NANPs to the assay.

We next inquired whether the presence of serum proteins interfered with ASGPR binding, potentially by occluding the ligand or forming a biomolecular corona that sterically shields the ligands. We thus evaluated the HepG2 cell association of TriGalNAc-functionalized NANPs under serum-free conditions. We did not observe cell association (Figure S6a), suggesting that serum protein adsorption is not the primary barrier to ASGPR-mediated uptake. We then examined whether insufficient ligand accessibility could contribute to the lack of binding by extending the ligand presentation from a 20mer to a 40mer dsDNA linker (corresponding to 7 and 14 nm from the NANP surface, respectively). However, increasing linker length did not alter the cell association (Figure S6b), indicating that limited ligand accessibility alone does not explain the absence of ASGPR engagement. Finally, we hypothesized that electrostatic repulsion between the negatively charged NANP and cell membrane may preclude TriGalNAc-ASGPR engagement. To test whether oligolysine-PEGylation could rescue cell association, we preincubated bare ICO or ICO-6T with oligolysine-PEG5K (K10-PEG5K) in a 1:1 N/P ratio, as previously established.^23^ Oligolysine-PEGylated NANPs were then incubated with HepG2 cells in serum-free media, but no cell association was observed (Figure S6c).

To investigate the behavior of TriGalNAc- and cholesterol-modified NANPs in other liver cell-type models, we conducted flow cytometry in the SK-HEP-1 system, where LDLR is expressed but ASGPR is not,^10^ and hypothesized that we would observe increased cell association for cholesterol-functionalized, but not TriGalNAc-functionalized NANPs. We observed ICO-*n*C NANPs associated with SK-HEP-1 cells in a similar valency-dependent manner as in HepG2 cells, but with lower overall association (Figure 2b), while we did not observe any cell association with TriGalNAc-functionalized NANPs (Figure 2c). For the J774A.1 model of Kupffer cells, we anticipated significant cell association independent of ligand functionalization, given well-established precedence that NANPs strongly associate with macrophages due to abundant scavenger receptor expression.^39,40,43,44^ We observed strong cell association for the nonmodified ICO, namely, 30–40-fold over the untreated groups, whereas incorporation of TriGalNAc or cholesterol increased this association. Interestingly, we did not observe a valency-dependent cell association as with the other systems, possibly due to saturation effects given the large background cell association.

After observing that cholesterol-functionalized NANPs exhibited a valency-dependent association with multiple liver cell types, we next sought to characterize the underlying mechanisms and pathways governing this association with hepatocytes. Confocal microscopy corroborated that ICO-6C was internalized by HepG2 cells, as increased intracellular fluorescence was observed in HepG2 cells treated with ICO-6C compared to bare ICO-treated or PBS-treated groups (Figures 2d and S7). Occasionally, larger and/or brighter AF647-positive structures consistent with multiparticle clusters were observed. We hypothesized that cholesterol-functionalized ICOs associate with hepatocytes via LDLR and SR-B1, similar to how lipoprotein-mimicking LNPs are internalized via LDLR. To test this hypothesis, we performed antibody blocking experiments to perturb specific putative ligand–receptor interactions. HepG2 cells were preincubated with monoclonal antibodies against LDLR and SR-B1, followed by exposure to either ICO or ICO-6C. Sterically blocking these receptors with monoclonal antibodies had no effect on the cellular association of ICO, which had negligible baseline association. In contrast, ICO-6C cell association was significantly reduced by each antibody blocking treatment (Figure 2e), supporting our hypothesis that LDLR and SR-B1 contributed to the association of cholesterol-modified ICOs with HepG2 cells.

To build on these findings, we then investigated whether and how ICO-6C was internalized using chemical perturbations. Specifically, we pretreated cells with Pitstop 2, cytochalasin D, dextran sulfate, polyinosinic acid (poly-I), methyl-β-cyclodextrin (MβCD), and colchicine to inhibit clathrin-mediated endocytosis, actin-dependent macropinocytosis, Class A scavenger receptors, Class A and B scavenger receptors, caveolin/lipid raft-associated pathways, and microtubule-dependent endocytic trafficking, respectively.^54^ As LDLR internalizes via clathrin-mediated endocytosis, we hypothesized that treating HepG2 cells with this well-established clathrin inhibitor^54,55^ would significantly reduce cell association. Pitstop 2 treatment decreased the association of ICO-6C with HepG2 cells, suggesting that clathrin-mediated endocytosis was the primary mechanism of internalization (Figure 2f). Treatment with MβCD also significantly reduced HepG2 association of ICO-6C, suggesting that membrane cholesterol plays a critical role in ICO-6C internalization; this may reflect disruption of cholesterol-dependent receptor dynamics as ICO-6C was preincubated with human serum prior to cell exposure but may also indicate impairment of any residual direct membrane insertion. This is consistent with prior findings that LDLR becomes confined to cholesterol-rich membrane domains and that cholesterol depletion alters receptor dynamics and reduces ligand binding and internalization efficiency.^56,57^ Our results suggested minimal involvement of the other endocytic pathways tested.

Together, these studies indicate that cholesterol-modified NANPs engage LDLR and SR-B1 receptors and internalize into hepatocytes via clathrin-mediated endocytosis and that this engagement and internalization can be modulated by engineering cholesterol valency. In contrast, TriGalNAc-modified NANPs failed to associate with hepatocytes despite confirmed ligand bioactivity, suggesting that the multivalent TriGalNAc display is insufficient for ASGPR engagement of NANPs. In nonparenchymal liver cell models, SK-HEP-1 and J774A.1, cholesterol-functionalized NANPs also exhibited moderate valency-dependent association with LSECs and strong macrophage association. These findings highlight how ligand chemistry, valency, and cell type together shape NANP association profiles with *in vitro* liver cell models.

### Controlling NANP Lipophilicity to Modulate Biomolecular Corona Formation and Hepatocyte Targeting

Given the programmable nature of ligand display with NANPs, we explored how this platform feature might be leveraged to modulate HepG2 association via biomolecular corona modulation. In this study, we examined the effects of lipid surface density, linker distance, and lipid chemistry (Figures 3a and S1b–f). Previous studies found that increased spacing of cholesterol on spherical NANPs heighten endothelial cell (HUVEC) association,^58^ and cholesterol spacing on DNA duplexes enhances membrane binding. We hypothesized that distributing cholesterol evenly across the surface of the NANP, as opposed to clustering it in a localized area, would enhance HepG2 association. To test this hypothesis, we compared cell association between NANPs displaying cholesterol clustered on a single vertex and symmetrically dispersed across the surface. ICOs with cholesterol moieties clustered on a single vertex (ICO-2C*, ICO-3C*, ICO-4C*, ICO-5C*) were compared to an ICO with cholesterol symmetrically dispersed throughout the NANP surface (ICO-5C) (Figure 3b). Flow cytometry revealed that symmetrically dispersed cholesterol led to a higher association with HepG2 cells compared to clustered configurations.

**Figure 3:**
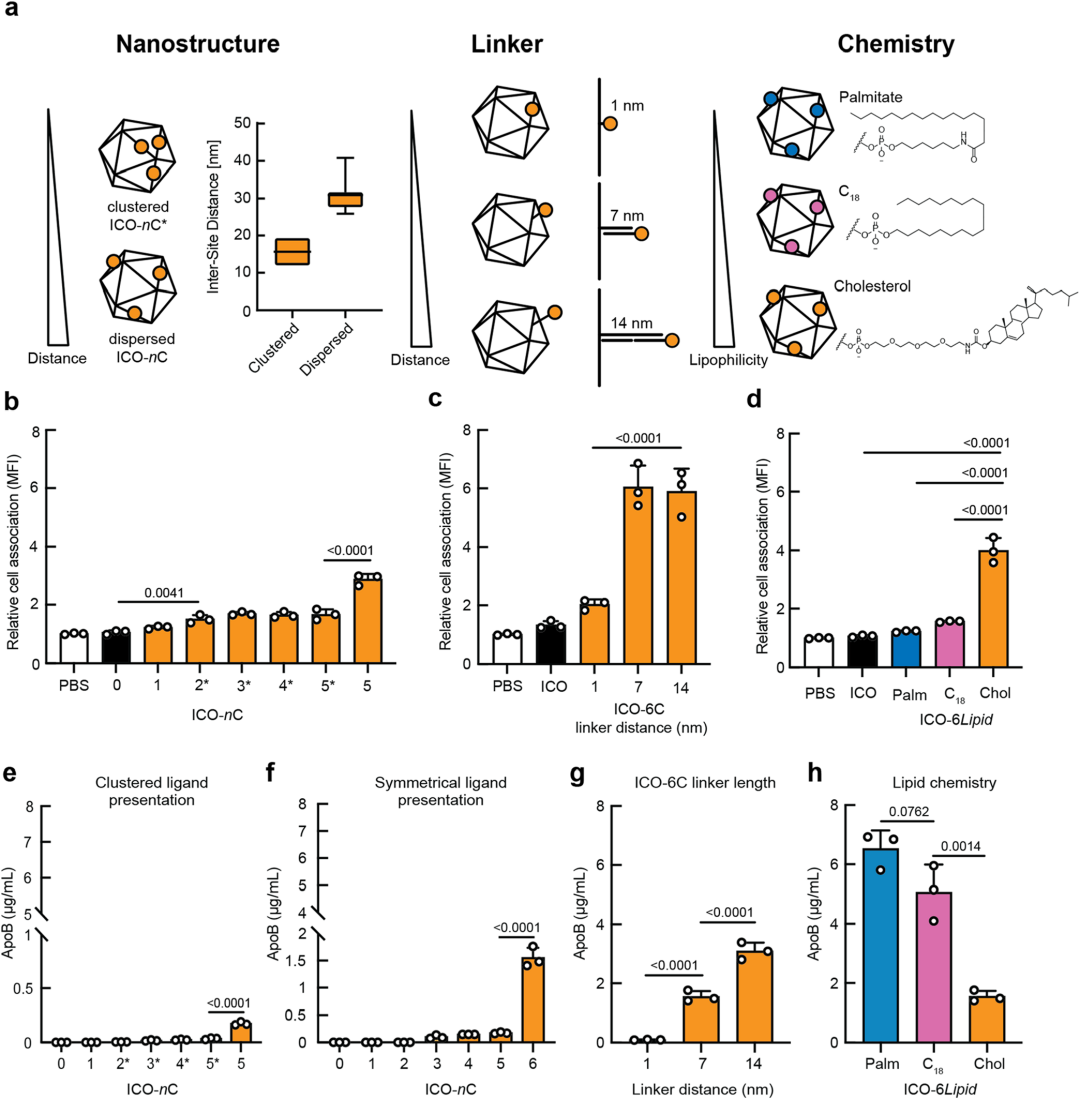
Investigating effects of lipid chemistry and nanoscale presentation on hepatocyte association and serum protein adsorption. (a) Schematic showing parameters modulated in this study: cholesterol clustering vs symmetrical dispersion, increasing linker display distance (1, 7, 14 nm), and comparison of lipid chemistries (palmitate, C_18_, cholesterol). Distances between functionalization sites (box and whiskers min. to max.) were calculated using ICO atomic models. (b) HepG2 association of ICOs functionalized with 1–5 copies of cholesterol clustered around a single vertex (ICO-1C to ICO-5C*) compared to symmetrical dispersion (ICO-5C). (c) HepG2 association of ICOs with 6 cholesterol molecules displayed at increasing distances from the NANP surface (1 nm, 7 nm, 14 nm). (d) HepG2 association of ICOs functionalized with palmitate (ICO-6PALM), C_18_ (ICO-6C_18_), or cholesterol (ICO-6C). (e–h) Quantification of ApoB recruitment by distinct ICO constructs after incubation in 55% human serum, measured by ELISA and reported as area under the curve (AUC). (e) ICO-*n*C constructs presenting 0–5 cholesterol, with * indicating constructs in which ligands are clustered on a single face of the NANP. (f) ICO-*n*C constructs presenting 0–6 cholesterol ligands distributed symmetrically across the NANP. (g) ICO constructs displaying 6 cholesterol ligands attached with varying linker lengths. (h) ICO constructs displaying 6 copies of three different lipid chemistries, each at a 7 nm linker. For panels (b–h), error bars indicate mean ± SD, *n* = 3 biological replicates. An ordinary one-way ANOVA with Tukey’s multiple comparisons test (α = 0.05) was conducted; *p*-values indicated.

Linker length is another critical parameter in ligand-based cell targeting that modulates multivalent binding by influencing avidity and spatial complementarity between ligands and receptors.^59^ Prior work demonstrated that positioning cholesterol further from DNA duplexes using a flexible tetraethylene glycol spacer enhances membrane attachment. Moreover, the linker length can influence cellular uptake by modulating ligand orientation and accessibility: excessively short linkers may limit flexibility and receptor engagement, while overly long linkers may lead to folding or shielding of the ligand.^59^ This phenomenon can be further understood in the context of the biomolecular corona, which forms upon exposure to serum and can sterically block surface-displayed ligands, particularly those positioned too close to the nanoparticle core.^4,37^ Similarly, PEGylation is known to reduce nonspecific interactions but can also shield targeting ligands from receptor recognition if the ligands are not sufficiently extended beyond the PEG.

Here, we hypothesized that short cholesterol linkers may be embedded within or sterically occluded by the biomolecular corona, limiting their ability to associate with apolipoproteins or engage with membrane receptors. Extending the cholesterol further from the NANP surface may therefore allow it to protrude beyond this shielding layer, increasing its bioavailability for productive interactions. To test this assumption, we investigated how the distance of cholesterol displayed from the NANP surface affected the hepatocyte association. We conjugated cholesterol either directly on the NANP at outward-facing 3′ termini of staples or extended from a 20mer or 40mer dsDNA linker extending from the same position, representing approximately 1, 7, and 14 nm linker lengths, respectively (Figure S1c). These lengths corresponded to either within, near the external surface of, or outside of the biomolecular corona, as approximated via TEM (Figure 1d). We observed that ICO-6C with a 1 nm linker had minimal association with the HepG2 cells, whereas the constructs using 7 and 14 nm linkers showed significantly increased association with HepG2 cells (Figure 3c). We found no significant difference between the 7 and 14 nm configurations, suggesting a critical length threshold exists to mediate this interaction.

Lipid chemistry is a critical parameter of cellular delivery efficiency in ASO and siRNA delivery;^13,14^ generally increased lipophilicity increases cell-membrane permeability.^13^ While cholesterol has been extensively explored for enhancing nucleic acid cell association, other lipids such as C_18_ ^14^ and palmitate^60^ are emerging as alternative chemistries for nucleic acid delivery. We hypothesized that these lipid chemistries may additionally modulate the association of hepatocytes with NANPs beyond the parameters explored above. To investigate the impact of lipid chemistry, we conjugated ICOs with lipids across a gradient of lipophilicity: cholesterol (ICO-6C), C_18_ (ICO-6C_18_), and palmitate (ICO-6 Palm) (Figures 3d, S1d and Supporting Note S1). At equivalent valences, cholesterol-functionalized ICOs exhibited the highest association in HepG2 cells, followed by C_18_- and then palmitate-functionalized ICOs (Figure 3b).

Having observed these design-specific HepG2 targeting properties, we next investigated the potential mechanisms underpinning these findings. We hypothesized that the HepG2 association was directly associated with lipoprotein recruitment within the biomolecular corona. Specifically, because apolipoproteins ApoB100 and ApoE are integral components of VLDLs, LDLs, and LNPs that enter hepatocytes through LDLR,^2–4^ we hypothesized that these NANPs recruited these apolipoproteins into their biomolecular corona, which facilitated their recognition and uptake via LDLR engagement. We recently reported an ELISA approach to probe the presence of individual proteins within a biomolecular corona of NANPs, which we adapted here to assess how NANP designs differentially recruit ApoB100 and ApoE.

ELISA results showed that increased cholesterol valency and dispersed cholesterol presentation significantly enhanced ApoB100 recruitment, consistent with the hypothesis that apolipoprotein adsorption facilitates hepatocyte uptake through receptor-mediated pathways (Figures 3e,f and S8a–d). Further, ApoB100 recruitment increased for longer linker lengths, with the NANP construct with 1 nm linker lengths being unable to recruit apolipoprotein (Figure 3g). Cholesterol-functionalized NANPs adsorbed the least ApoB100 compared with C_18_- and palmitate-functionalized NANPs, which is inconsistent with our flow cytometry results that showed cholesterol, followed by C_18_ and palmitate, led to the highest cell association (Figure 3d,h). This observation suggests that ApoB100 recruitment is not the sole or primary factor that dictates cell association, which may occur through distinct mechanisms such as direct cell-membrane association of the NANP. We also investigated ApoE recruitment. We did not observe ApoE recruitment in any of the NANP constructs, potentially due to low affinity for the lipid-modified ICOs, or its relatively low abundance of 5–8 mg/dl present in human serum (Figure S8e).

Collectively, these results demonstrated that engineering the lipophilicity of NANPs can modulate cell targeting. For HepG2 targeting through an LDLR-based mechanism, we identified that symmetrical, dispersed ligands displayed several nanometer linker lengths above a critical threshold, and highly lipophilic lipid chemistry, enhance cell association through mechanisms involving ApoB100 recruitment, LDLR and SR-B1 engagement, and clathrin-mediated endocytosis.

### Benchmarking Cell Targeting Capabilities of NANP, Conjugate, and LNP Systems

To assess the capability of ICO origami NANPs to deliver siRNA to liver cells, we conjugated siRNAs targeting ALDH2 (Table S4),^61^ a housekeeping gene highly expressed in hepatocytes, to ICO-6C, which led to the greatest HepG2 association for bare particles (Figures 1a and S9). We benchmarked its performance against two state-of-the-art delivery platforms: a TriGalNAc-siRNA conjugate similar in formulation to *nedosiran*, an siRNA drug made using Dicerna’s GalXC technology, and an LNP-siRNA formulation similar to *patisiran*, Alnylam’s LNP-siRNA delivery system. We assessed their cell association across the three previously mentioned major liver cell types and evaluated their RNAi potency in HepG2 cells.

To assess whether incorporation of siRNAs into our NANPs altered their cell association with respect to bare particles, we tested different siRNA valences (2, 6, 12, and 24 siRNAs per NANP) in HepG2 cells by flow cytometry and found that increasing the siRNA valency of cholesterol-modified ICOs decreased cell association monotonically (Figure S10). To balance loading ratios and cell targeting capabilities, we selected ICO-6C-12siRNA to benchmark against the conjugate and LNP systems. The sense strands of the siRNAs were modified with AF647 to ensure consistent dosing and accurately benchmark siRNA delivery efficiency. Flow cytometry analysis in HepG2 cells revealed that at 1 h, the ICO-6C-12siRNA exhibited significantly higher cellular association than both the TriGalNAc-siRNA conjugate and LNP-siRNA (Figure 4a). However, upon extending the incubation time up to 4 h to probe early internalization kinetics while minimizing downstream intracellular processing, LNP-siRNA showed a time-dependent increase in cell association, surpassing ICO-6C-12siRNA. This is consistent with *patisiran*’s design, which incorporates a stealth PEG coating that gradually disassociates to facilitate ApoE recruitment and LDLR-mediated endocytosis. Importantly, TriGalNAc-siRNA also did not bind to HepG2 cells, even when incubated at considerably higher concentrations (Figure 4a). In contrast, a dsDNA of similar size conjugated to TriGalNAc showed binding to HepG2 cells at the same concentrations (Figure S5b).

**Figure 4:**
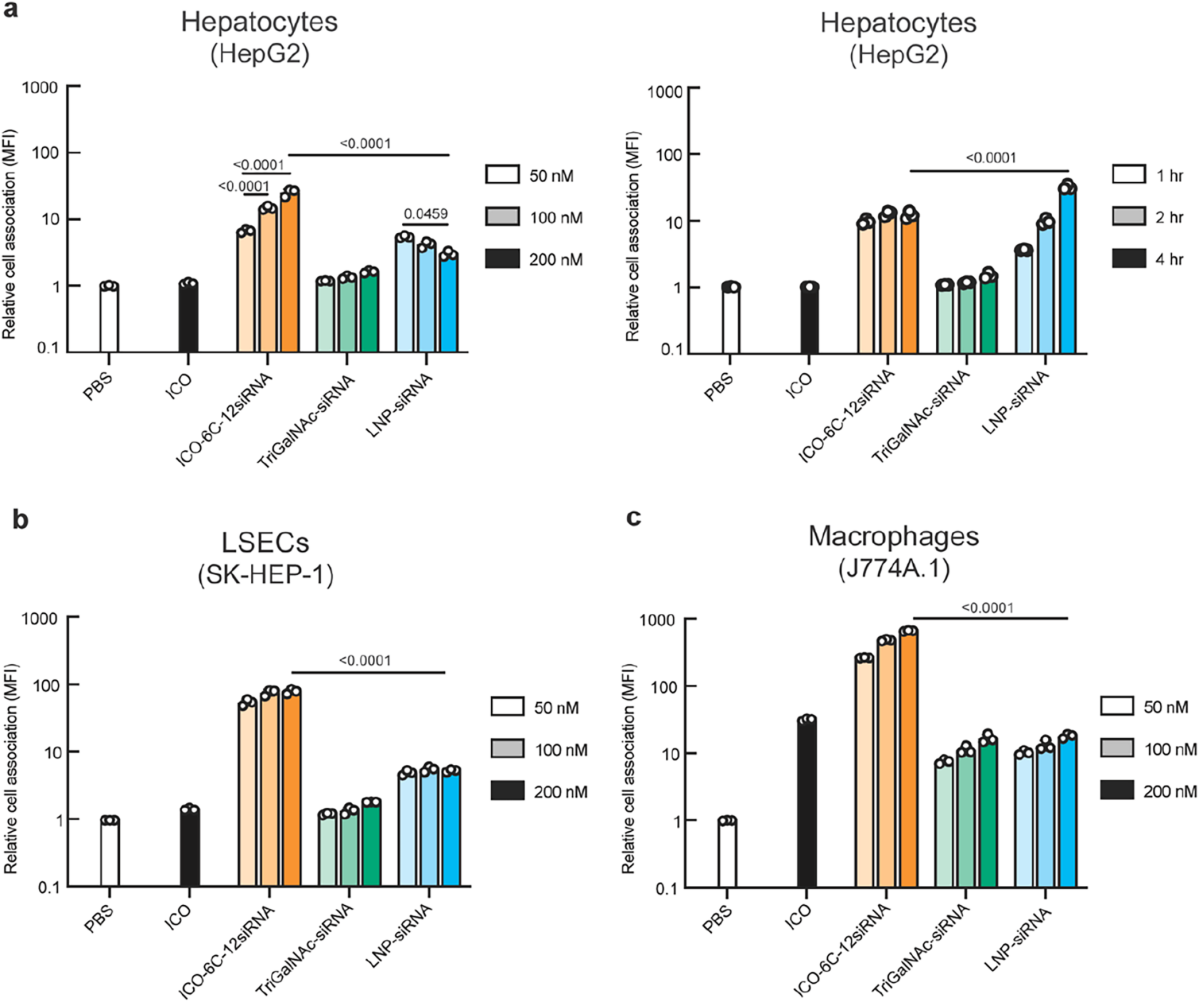
Benchmarking NANP cell association against clinical siRNA delivery systems. Relative cell association of siRNA-functionalized NANPs (ICO-6C-12siRNA), TriGalNAc-siRNA conjugate, and LNP-siRNA in HepG2 (a, left), SK-HEP-1 (b), and J774A.1 (c) cells. Association was quantified by flow cytometry using AF647-labeled siRNA strands over a dose range (50, 100, and 200 nM). PBS and ICO (200 nM) were assessed as negative controls. These constructs were also assessed in HepG2 cells at 50 nM for different incubation times (a, right) to compare the association kinetics. For panels (a–c), error bars indicate mean ± SD, *n* = 3 biological replicates. An ordinary two-way ANOVA with Tukey’s multiple comparisons test (α = 0.05) was conducted; *p*-values indicated.

In SK-HEP-1 cells, TriGalNAc-siRNA showed minimal association, in line with its specificity for the ASGPR receptor, which is largely absent in this cell type (Figure 4b). In contrast, ICO-6C-12siRNA exhibited robust association, likely due to interactions with LDLR and SR-B1.^37^ In addition to LDLR and SR-B1, LSECs express other scavenger receptors such as stabilin-1 and stabilin-2, which are known to internalize phosphorothioate-modified oligonucleotides.^62^ The presence of phosphorothioate-modified siRNAs on ICO-6C-12siRNA may therefore have contributed to the elevated level of SK-HEP-1 association observed, potentially through recognition by these scavenger receptors. The LNP formulation also showed high association with SK-HEP-1 cells, consistent with prior reports showing that ionizable lipid nanoparticles were readily internalized by various liver cell types through a combination of size-dependent permeability and receptor-mediated uptake mechanisms, including LDLR and scavenger receptor pathways.^2,4^

In J774A.1 cells, all three delivery systems exhibited substantial association (Figure 4c), with ICO-6C-12siRNA demonstrating the highest association. This result was consistent with other reports that Kupffer cells can sequester both conjugate- and LNP-based delivery systems.^4,39^ The high macrophage association observed is consistent with the established role of macrophages in mediating DNA clearance^52^ and may reflect enhanced recognition of more structurally complex 3D DNA architectures^43^ with higher DNA density.^44^

Collectively, our benchmarking analysis demonstrated that lipophilic NANP-siRNAs achieved higher hepatocyte, endothelial cell, and macrophage association than did the LNP-siRNA and conjugate-siRNA systems at early time points. However, although ICO-6C-12siRNA association plateaued at early exposure times, LNP-siRNAs exhibited delayed yet increasing association with extended exposure, highlighting fundamental differences in the cellular uptake kinetics of these different delivery systems.

### Benchmarking RNAi Capabilities of NANP, Conjugate, and LNP Delivery Systems

We next compared these systems’ abilities to mediate RNAi by assessing ALDH2 knockdown in HepG2 cells via RT-qPCR, comparing ICO-6C-12siRNA with TriGalNAc-siRNA and LNP-siRNA (Figure S11) under conditions with and without transfection reagent (Figure S12a,b). We observed ALDH2 knockdown in the presence of the transfection reagent for ICO and ICO-6C conjugated to siRNA, as well as for the TriGalNAc-siRNA conjugate, indicating that the siRNA remains functional after formulation with NANPs. However, we did not observe knockdown with either the ICO-6C-siRNA or TriGalNAc-siRNA in the absence of the transfection reagent. In contrast, the LNP-siRNA formulation achieved high-efficiency RNAi autonomously, without the need for transfection (Figure S12a). The failure of the TriGalNAc-siRNA conjugate to silence ALDH2 under autonomous conditions is consistent with our earlier findings that these TriGalNAc-siRNAs exhibited minimal association with HepG2 cells (Figure 4a). Importantly, the TriGalNAc-siRNA conjugate did lead to robust RNAi *in vivo* after intravenous administration (Figure S12c), suggesting that the lack of RNAi observed *in vitro* is likely due to a model artifact rooted in the HepG2 cell line.

### Nonhepatocytic Modulation of NANP Cell Association through Lipid Modifications

While lipid-based ligands were initially explored to enable siRNA delivery to the liver, these ligands are now increasingly explored as avenues to realize delivery in extrahepatic organs and systems.^13^ We hypothesized that similar lipid-based design strategies explored in this study to achieve hepatocyte cell model targeting may facilitate the targeting of other cell systems. To explore this potential, we investigated cell association and uptake of various lipophilic NANPs in Neuro-2a (N2a) cells, a murine neuroblastoma cell line used as a first-pass cellular system to model neurons in targeting studies.^63^

We hypothesized that cell association would be valency-, spacing-, and chemistry-dependent in this model, generalizing our results from the liver model systems. Using the same NANP design, we conjugated 1–6 lipid moieties of either cholesterol, C_18_, or palmitate. Each lipid was either symmetrically dispersed or clustered at a single vertex of the NANP (Figure S1). These constructs were incubated with N2a cells across a 0.1–100 nM dose range, and cell association was quantified using a high-throughput fluorescence plate reader assay. Importantly, to mimic physiological conditions, these NANPs were not preincubated in serum as cerebrospinal fluid and interstitial fluid of the CNS parenchyma are significantly lower in protein abundances than blood.^64^

Across all lipid types, we observed dose-dependent and valency-dependent increases in association with N2a cells (Figure 5a–c). Cholesterol-functionalized ICOs consistently exhibited the highest levels of association, followed by C_18_ and palmitate, a trend similar to that observed in the HepG2 model. For cholesterol, clustering the lipids provided no added benefit over dispersed presentation. However, for C_18_ and palmitate, clustering enhanced cell association compared with dispersed arrangements at the same copy number. This suggests that nanostructuring of less hydrophobic lipids may improve their effective presentation or membrane interaction, compensating for their lower intrinsic affinity. Hence, the effects of lipophilicity and lipid nanostructuring on cell association of NANPs clearly differ based on the cell model under investigation.

**Figure 5:**
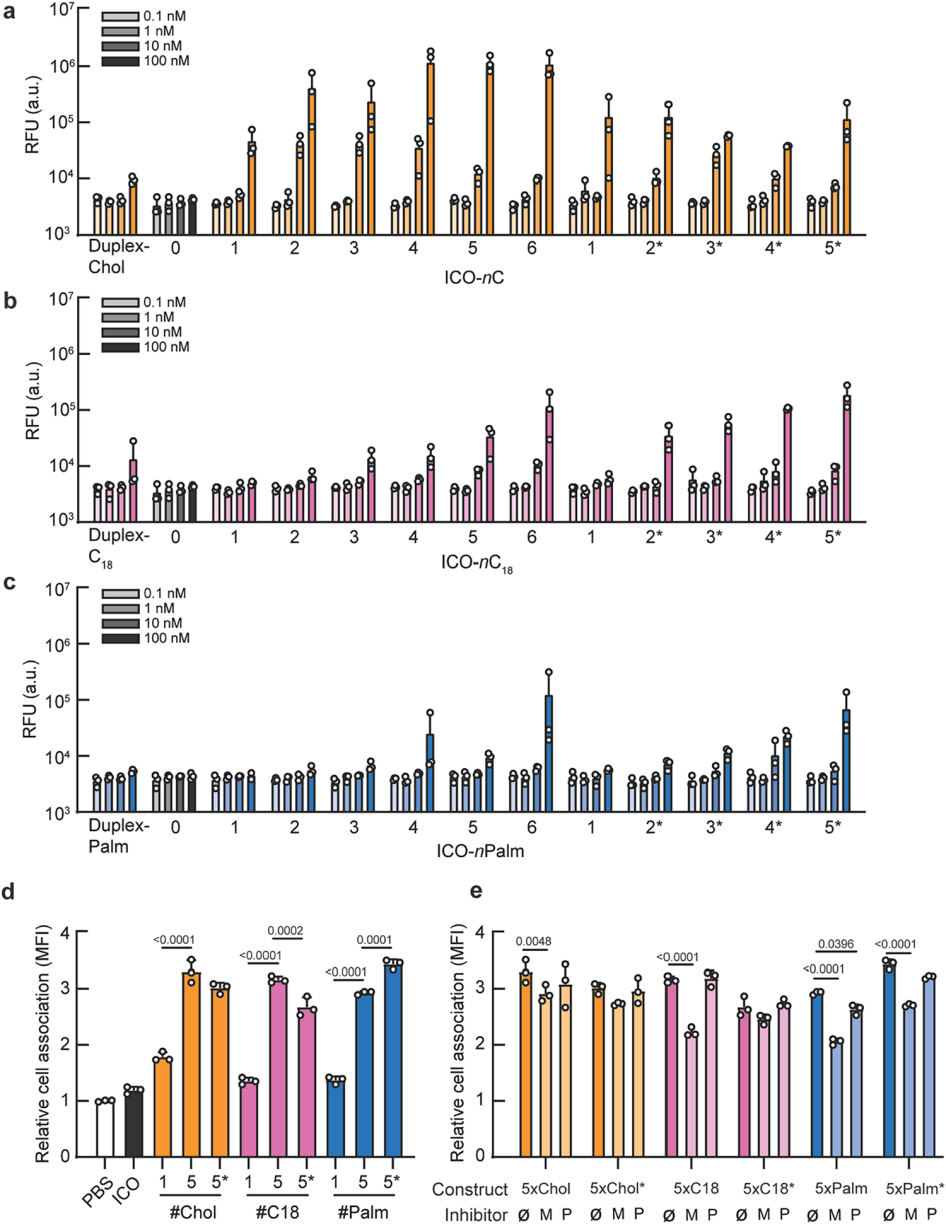
Controlling NANP lipid chemistry, valency, and spatial arrangement modulates association with neuronal cells. Association of a NANP library with N2a cells, when conjugated to 1–6, dispersed or clustered, moieties of cholesterol (a), C_18_ (b), or palmitate (c). Cell association was quantified via fluorescence (RFU) using a plate reader. For each construct, cells were treated at log-fold doses: 0.1, 1, 10, and 100 nM. (d) Cellular association of a subset of the NANP library with N2a cells analyzed via flow cytometry. (e) Effects of membrane cholesterol depletion (MβCD) and clathrin inhibition (Pitstop 2) on N2a cellular association of ICOs were quantified with flow cytometry. For panels (a–e), error bars indicate mean ± SD, *n* = 3 biological replicates. For panels (d) and (e), an ordinary two-way ANOVA with Tukey’s multiple comparisons test (α = 0.05) was conducted; *p*-values indicated. Ø: no inhibitor; M: MβCD; P: Pitstop 2.

We next focused on constructs with the highest levels of cell association to probe the underlying mechanisms. Cells were seeded in 48-well plates and incubated for 1.5 h with ICOs functionalized with 5 copies of cholesterol, C_18_, or palmitate, either dispersed across the structure or clustered at a single vertex. Cell association was first quantified via flow cytometry following incubation in serum-free medium (Figure 5d). To investigate internalization mechanisms, we drew upon previous studies of cholesterol-conjugated oligonucleotides, which suggest a two-step uptake process: initial membrane anchoring via lipid–membrane interactions followed by receptor-mediated endocytosis. To test this mechanism, we therefore pretreated N2a cells with either MβCD to disrupt lipid rafts and caveolin-mediated uptake or Pitstop 2 to inhibit clathrin-mediated endocytosis (Figure 5e). These inhibitors allowed us to probe the contributions of membrane lipid domains and endocytic pathways driving NANP uptake.

Flow cytometry analysis revealed that MβCD significantly reduced cell association for cholesterol-, C_18_-, and palmitate-functionalized NANPs, suggesting the involvement of lipid raft-mediated interactions. Pitstop 2 treatment led to a much more moderate but statistically significant reduction in association, consistent with clathrin-dependent internalization as a secondary uptake mechanism. In contrast, neither inhibitor significantly affected the baseline association of unmodified ICOs. These data suggest that the enhanced uptake observed with lipid-functionalized NANPs in N2a cells relies on lipid–membrane interactions and, to a lesser extent, receptor-mediated endocytosis, with dependence on these pathways varying by the lipid type and nanoscale organization.

Together, these findings reinforced the importance of lipid chemistry, valency, and spatial arrangement in controlling NANP cell interactions. While cholesterol enabled robust association, largely irrespective of structural arrangement, less hydrophobic lipids, such as C_18_ and palmitate, benefited from clustering. These results demonstrated that the lipidation strategy investigated in hepatic models are extensible to nonhepatic cell types including N2a cells. More broadly, they highlight the potential utility of NANPs as a programmable platform for targeted nucleic acid delivery across diverse tissues.

## Conclusion

We investigated the roles of composition and spatial presentation of lipids on cell association and the uptake of NANPs with and without siRNA cargo. Using an icosahedral NANP as a model system, we explored lipids of varying lipophilicity—cholesterol, C_18_, and palmitate—and systematically varied lipid chemistry, valency, density, and linker distance to modulate cellular association in representative model liver cell lines. We also cross-examined these findings in a model neuronal cell line. Specifically, when presented at stoichiometrically defined valences from the NANP surface, cholesterol conjugation significantly increased cellular association across HepG2, SK-HEP-1, and J774A.1 cells. Notably, bare ICOs exhibited minimal association with HepG2 and SK-HEP-1 cells but substantial uptake by J774A.1 macrophages. This high macrophage association is likely due to their innate phagocytic activity and proclivity to clear foreign genetic material, as well as their endogenous role in scavenging cell-free DNA.^43,44,52^ Ligand modification further enhanced uptake, potentially by increasing recognition through scavenger receptors or by promoting interactions with lipid- or glycan-binding proteins involved in receptor-mediated endocytosis. PEGylation used in combination with targeting ligands provides a future strategy to evade macrophages while targeting specific cells, although this strategy may be more applicable to active rather than passive targeting strategies.^23^

LDLR and SR-B1, while highly expressed in hepatocytes, are also present on endothelial cells and macrophages, which may contribute to the broader association profile observed with cholesterol-modified NANPs. While observed only in the *in vitro* and model cell lines, the observed association with SK-HEP-1 cells raises potential safety concerns, as endothelial uptake has been implicated in liver toxicity with LNP-based delivery systems. Further studies with primary cells and *in vivo* cells will be needed to assess the implications of this uptake pattern for NANP safety and specificity. Mechanistic studies including chemical inhibitors, confocal microscopy, antibody blocking, and ELISA support a model in which cholesterol-modified NANPs associated with lipoproteins in serum, engaged with LDLR and SR-B1, and underwent receptor-mediated endocytosis through clathrin-mediated pathways, in addition to other internalization pathways. This is similar to how FDA-approved LNP *patisiran* endogenously targets hepatocytes to deliver siRNAs. Notably, confocal imaging occasionally revealed larger and/or brighter AF647-positive structures indicating multiparticle clusters with atypical membrane interactions. Because cholesterol itself can partition into lipid bilayers, it is plausible that a fraction of cholesterol NANPs may exhibit an increased plasma membrane association via hydrophobic insertion, which could contribute to the observed cell-surface and intracellular signal, alongside LDLR- and SR-B1-dependent uptake pathways.

Interestingly, we found that dispersing cholesterol across the NANP surface symmetrically yielded higher HepG2 association than clustering lipids on a single vertex for ICO, consistent with prior reports highlighting the importance of ligand accessibility.^58,59^ Extending cholesterol away from the NANP surface improved uptake, supporting a model in which there exists a critical display length away from the surface in which cholesterol is bioavailable for the recruitment of lipoproteins and engagement with receptors. Importantly, this critical length may be different for active and passive targeting strategies and highlights the significance of individual optimization of linker design for each targeting strategy employed in a delivery system. By varying lipid chemistries with identical valences, we found cholesterol led to more cell association than C_18_ and palmitate, in agreement with their theoretical hydrophobicity. While our observation that more lipophilic lipids enhance HepG2 association is consistent with *in vivo* findings that more hydrophobic siRNAs demonstrate higher liver accumulation, experimentation with primary cells and *in vivo* will be needed to corroborate this finding.^13^ Notably, ELISA demonstrated that the less hydrophobic lipids resulted in larger ApoB recruitment, suggesting total ApoB recruitment is only partially correlated with cell association and that other factors such as lipid–lipoprotein affinity and direct insertion that may be modulated by lipid orientation could influence downstream LDLR recognition and hepatocytic uptake.

Surprisingly, in contrast to the cholesterol-targeting strategy, TriGalNAc-functionalized NANPs did not associate with HepG2 cells, despite evidence of ligand bioactivity from biochemical binding assays and cell studies with smaller nucleic acid nanostructures, namely, the Holliday junction and tetrahedron employed in this study. This suggested that factors such as size, charge, geometry, and/or steric hindrance from the NANP might impair ligand–receptor interactions at the cell surface. Notably, P-VLPs of similar size^10^ and larger LNPs^2^ can internalize via TriGalNAc-ASGPR, suggesting that the size limits observed with alternative delivery systems may not directly extend to highly charged NANP systems that may also be subject to competitive off-target scavenger receptor interactions. Although we evaluated NANP association under serum-free conditions to exclude potential effects from protein corona formation and explored charge-shielding through oligolysine–PEG treatment, neither strategy improved HepG2 association, implying that serum protein interference and electrostatic effects were unlikely to fully account for the observed lack of binding. Given the lack of binding observed with the TriGalNAc-siRNA positive control, despite the observed HepG2 association with a similarly TriGalNAc-conjugated and AF647-labeled DNA duplex, artifacts of the HepG2 cell model itself may be the cause. This is suggested by TriGalNAc’s functional behavior *in vivo* as a hepatocyte targeting ligand, as demonstrated in this study and in others.^65^ Limitations of the HepG2 model may be due to insufficient receptor expression levels or deficient receptor-mediated endocytosis and recycling, despite the observed association with TriGalNAc-Holliday junctions. Follow-up studies investigating these NANPs in primary human and murine hepatocytes, as was performed during the development of GalNAc conjugate technologies,^11^ should provide additional insight, but are beyond the scope of the present study.

To understand NANP performance as cell delivery vectors using the lipids examined, given that HepG2 association was not observed using TriGalNAc, we benchmarked them against clinically relevant TriGalNAc-siRNA and LNP-siRNA formulations. In flow cytometry studies, ICO-6C-12siRNA exhibited a rapid and robust association with hepatocytes, endothelial cells, and Kupffer cells. In time-series studies, LNP association with HepG2s increased over time, consistent with delayed PEG deshielding and ApoE-mediated uptake. The differences in cell association kinetics between NANP, conjugate, and LNP systems affirm that the cellular uptake kinetics of these materials are distinct, as anticipated, which might offer the potential for differential distribution properties *in vivo*. Pharmacokinetic studies in animal models comparing these technologies are therefore of highest importance, together with primary cell assays, to build on the foundational studies performed here in model cell lines. Notwithstanding, our findings provide valuable benchmarks for how cholesterol-functionalized NANPs interact with diverse liver cell types, relative to established delivery technologies. Moving forward, leveraging the modular programmability of NANPs to enhance cell-type selectivity and reduce off-target uptake in nonhepatocyte populations such as LSECs and macrophages via passivation strategies such as PEGylation may be useful for achieving hepatocyte-specific delivery.^23,46^ Further, investigating whether passivation strategies like PEGylation in the context of both active (e.g., TriGalNAc) and passive (e.g., cholesterol) targeting ligands will maintain the requisite biomolecular corona and receptor interactions while removing undesired off-target binding. In addition, covalent versus noncovalent PEG may be important to compare.

Despite their association with and internalization by HepG2 cells, cholesterol-functionalized NANPs did not induce gene knockdown in the absence of additional transfection reagents. This may potentially be due to a failure to escape endosomes needed to deliver the siRNAs to the cytosol, which warrants future studies together with siRNA release from the NANP itself within endosomes.^7^ Unlike LNPs, these NANPs have no components designed to disrupt or modulate the endosomal membrane. While incorporation of chemical or structural features that promote endosomal disruption may be important to realize autonomous gene silencing with NANPs, endosomal leaking or receptor recycling may alternatively be sufficient for siRNA release and silencing. Notably, when transfected into HepG2 cells, NANP-siRNA formulations decreased transcript levels, suggesting the siRNA was indeed able to release from the NANP, potentially via intracellular nucleases degrading the nonchemically modified NANP structure. Given that the TriGalNAc-siRNA positive control also failed to associate with HepG2 cells and induce RNAi in the absence of the transfection reagent, the intrinsic properties of HepG2 cells, as noted above, may contribute to the observed lack of silencing.

Finally, we investigated whether principles of engineering NANP lipophilicity observed in liver cell lines were extensible to nonhepatic cells. In a neuronal cell model, we observed that increasing the lipid valency and hydrophobicity also enhanced NANP association. Chemical inhibition studies suggested that the mechanisms of internalization were distinct from those in the hepatocyte cell model. Notably, these distinct cell associations occurred without biomolecular corona interactions, suggesting that lipophilic modulation of NANPs might offer a more general strategy to pursue both passive and active targeting strategies, which remains to be explored *in vitro* and *in vivo*.

Taken together, our findings help elucidate the roles of NANP surface properties on interactions with cell line models representative of hepatocytes, liver endothelial cells, and macrophages, and in the nonhepatic context of model neurons. These results provide foundational insight into the mechanisms governing NANP receptor targeting, internalization, and cargo delivery specifically, which may generalize to other nanomaterials and nanoparticles. While substantial further work is needed to understand siRNA delivery using NANPs including tissue- and cell-level biodistribution and RNAi, the programmability and modularity of NANPs offer important potential for tailoring delivery for distinct therapeutic applications.

## Methods

### Ethics Statement

Animals were housed and handled in the Association for Assessment and Accreditation of Laboratory Animal Care (AAALAC)-accredited facilities with experimental methods as specifically approved by the Institutional Animal Care and Use Committees at MIT (MIT CAC Protocol #2303000478).

### Materials

Magnesium chloride (#M2670), 10× TAE (#574797), drop dialysis membranes (#VSWP02500), PBS containing 0.05% Tween-20 (#524653) and 4 mL, 100 kDa (#UFC8010) Amicon filters, cytochalasin D (C2618—200UL), and poly-I (P4154—10MG) were acquired from MilliporeSigma. Nuclease-free water (#11-05-01-04) was acquired from IDT. PBS (#21-040-CM) was acquired from Corning. Oligonucleotide synthesis reagents and consumables were acquired from Glen Research. Bovine serum albumin (#37525), Ultra TMB-ELISA substrate (#34021), Wheat Germ Agglutinin Alexa Fluor 488 (#W11261), Alexa Fluor 647 NHS Ester (#A20006), PBS containing magnesium and calcium (#14040133), poly-d-lysine (#A3890401), SYBR Safe (#S33102), High-Capacity cDNA Reverse Transcription Kit (#4374966), Taqman probes for human ALDH2 FAM (#4331182), human HRPT VIC (#4331182), mouse ALDH2 FAM (#4331182), mouse HRPT VIC (#4331182), cell scrapers (#179693), methyl-β-cyclodextrin (J66847.06), and colchicine (J61072.ME) were acquired from ThermoFisher Scientific. Oligolysine-PEG was acquired from Alamanda Polymers (#mPEG5K-*b*-PLKC10). Molecular-biology-grade agarose (#87004-980), sodium chloride (#BDH9286), HPLC-grade acetonitrile (#BDH83639.402), water (#BDH23595.400), and Triton-X-144 (#M144) were acquired from VWR. One kb Plus DNA Ladder (#N0550S) and 6× Gel Loading Dye (#B7025S) were acquired from New England BioLabs. Paraformaldehyde (#15710), TEM grids (#CF200H-CU-50), and uranyl acetate (#22400) were acquired from Electron Microscopy Sciences. Recombinant ASGR1 (AS1-H5243) was acquired from ACROBiosystems.

HepG2 (#HB-8065), SK-HEP-1 (HTB-52), J774A.1 (TIB-67), and Neuro-2A cells (CCL-131), DMEM medium (#30-2002), fetal bovine serum (#30-2020), penicillin–streptomycin (#30-2300), and 0.05% Trypsin-EDTA (1×) (ATCC, 30-2101) were purchased from ATCC. 2× Yeast Extract Tryptone Medium (2× YT) dry powder (Y2377-250G), carbenicillin (#C3416-1G), chloramphenicol (#C0378-25G), sulfuric acid (#258105-500 ML-PC), dextran sulfate sodium salt (42876-5G), PEG8000 flakes (#89510-1KG-F), and sterile-filtered human male plasma Type AB human serum (#H4522) were purchased from Sigma-Aldrich. Zombie UV Live–Dead stain (#423107), antihuman CD36L1 (SCARB1, SR-BI) antibody (#**363201**), antihuman CD204 antibody (#**371905**), and 96-well Nunc Maxisorp plates were purchased from BioLegend Inc. Antihuman antibodies against LDLR (#MAB2148), ASGPR (#FAB43941R), apolipoprotein B (#MAB4124-SP), and apolipoprotein E (#MAB41444-SP) were purchased from R&D Systems. Pitstop 2 (ab120687) and DAPI staining solution (ab228549) were purchased from Abcam. Twelve well chamber removable microscopy slides (#81201) were purchased from ibidi. Prolong Gold Antifade Mountant (P36934), Alexa Fluor 647 azide, and triethylammonium salt (#A10277) were purchased from Invitrogen. Horseradish peroxidase (HRP)-conjugated goat antimouse IgG secondary antibody (#1706516), iScript RT-qPCR Sample Preparation Reagent (#1708899) was purchased from BioRad. HiSpeed EndoFree Giga Kit (#1054575) and RNeasy Plus Mini Kit (#74136) were acquired from Qiagen. Endosafe LAL-based Gel-Clot Endotoxin Test (#R135) was purchased from Charles River Laboratories. Trivalent β-GalNAc-PEG3-azide (#MV10020) was purchased from Sussexresearch Laboratories. Liver tissues were homogenized with Spex SamplePrep using the Geno/Grinder in 4 mL vials from OPS Diagnostics using 5/16″ stainless steel balls.

### Equipment

Custom-length DNA scaffolds were produced by using an Eppendorf Innova 44i Shaker-Incubator. Oligonucleotide synthesis was conducted on a Biolytic Dr. Oligo 192c Synthesizer. Oligonucleotide purification was performed on a Waters ACQUITY liquid chromatography system with an XBridge C18-BEH 130 Å column. Oligonucleotides were dried by using a ThermoFisher SPD300 rotary SpeedVac system, and DNA concentrations were measured using a ThermoFisher Scientific NanoDrop 2000. Liquid handling for NANP staple pools was conducted using a Formulatrix FLO i8. NANPs were self-assembled using an Eppendorf Thermomixer C. Agarose gel electrophoresis (AGE) was performed using BioRad equipment. Gel imaging occurred on a Typhoon FLA 7000. Dynamic light scattering (DLS) was performed using a Malvern ZetaSizer and Wyatt Dyna Pro Plate reader (AF647-labeled NANPs). Transmission electron microscopy (TEM) was conducted by using an FEI Tecnai transmission electron microscope. All flow cytometry analyses were conducted on a FACS LSR Fortessa High Throughput Sampler II. Confocal microscopy was conducted on a Leica SP8 laser scanning microscope with a 63× oil objective. A Tecan Spark plate reader was used for measuring ELISA absorbance as well as cellular association in Neuro-2A cells. cDNA synthesis was performed by using a BioRad T100 Thermal Cycler. Quantitative PCR (qPCR) analysis was done on a Roche Light Cycler 480 II Real-time PCR machine with a 384-well plate format. Cell counting was done using the Cellometer Auto 2000 instrument from Nexcelom Bioscience.

### Scaffold Production

Custom scaffold DNA sequence of length 3120 nt for ICO52 NANPs was biologically produced as previously.^66^ In brief, SS30*Escherichia coli*was cotransformed with M13 cP helper plasmid (originally provided as a gift from Andrew Bradbury, Los Alamos National Laboratories) and miniphage constituting only a f1 origin, β-lactamase-encoding antibiotic resistance gene to produce the corresponding circular single-stranded DNA sequence. Precultures were prepared from a discrete colony of transformed cells and grown overnight in 2× YT media supplemented with 100 μg/mL carbenicillin and 15 μg/mL chloramphenicol at 37 °C. The following day, precultures were transferred at an optical density (OD) of 0.05–0.1 to 1 L Erlenmeyer flasks containing 2× YT media supplemented with 100 μg/mL carbenicillin, 15 μg/mL chloramphenicol, and 5 mM MgCl_2_ solution and grown at 37 °C and 200 rpm for 8 h or an OD ∼8.0–10.*E. coli*cells were pelleted once by centrifugation at 4 °C, and the supernatant was collected in a separate container containing 3% (w/v) PEG8000 flakes and 3% (w/v) NaCl and incubated overnight under gentle shaking at 4 °C for phage precipitation. Next day, the phages are harvested by centrifugation at 4000*g* for 1 h at room temperature and subsequently lysed to isolate and purify the custom-length DNA using the Qiagen Endofree Gigaprep kit protocol with a few modifications: proteinase K was added at 400 ug per liter culture to P1 buffer followed by incubation at 37 °C for 1 h, addition of P2 buffer, and incubation at 70 °C for 10 min. Following purification of circular ssDNA, residual endotoxins were removed using Triton-X-144. Endotoxin levels were measured using the LAL-based Gel-Clot Endotoxin Test. DNA scaffold purity was validated by AGE.

### Oligonucleotide Synthesis and Modification

Modified oligonucleotides were fabricated in-house. A (#10-1000), C (#10-1010), G (#10- 687 1020), T (#10-1030), cholesteryl-TEG (#10-1976), stearyl (#10-1979), palmitate (#10-1978), and DBCO-TEG (#10-1941) phosphoramidites and ancillary synthesis reagents were acquired from Glen Research and used following the manufacturer’s recommendations. Oligonucleotides were synthesized on a Biolytic Dr. Oligo 192c oligonucleotide synthesizer, at a 200 nmol scale with CPG 1000 Å standard base supports (#20-2001, #20-2011, #20-2021, #20-2031) in normal mode under nitrogen. Synthesis success and yield was observed by monitoring the penultimate trityl-cleavage. Following the synthesis, oligonucleotides were cleaved and deprotected under pressure at 55 °C for 2 h in 30% ammonium hydroxide (ThermoFisher Scientific, cat# 423305000). The cleaved strands were desalted with acetonitrile and eluted in nuclease-free water. Modified oligonucleotides were then purified with HPLC with the following reverse-phase protocol: 0–1 min: 90:10 A:B; 1–11 min: 90:10 A:B to 55:45 A:B over a linear gradient; 11–16 min: 55:45 A:B to 20:80 A:B over a linear gradient; 16–20 min: 20:80 A:B to 90:10 A:B over a step gradient (A: 0.1 M aqueous triethylammonium acetate; B: acetonitrile). Oligonucleotides were dried under vacuum and then resuspended in nuclease-free water for further analysis and use. Oligonucleotide concentrations and yields were determined via UV–vis measurements. Two equivalents of TriGalNAc-azide or AF647-azide were incubated with DBCO-oligonucleotides at 37 °C for 48 h before HPLC purification using the reverse-phase protocol above, dried under vacuum, and resuspended in nuclease-free water to yield TriGalNAc-oligos for hepatocyte targeting, and AF647-oligos for tracking NANPs in flow cytometry and confocal microscopy experiments. Yields were determined by UV–vis measurements, and the purity was confirmed by HPLC. To track the siRNA in flow cytometry experiments, the sense strand (SS) of siRNAs was functionalized with an Alexa Fluor 647 dye using NHS chemistry. Briefly, the SS oligonucleotides were reacted with 10-fold excess AF647-NHS ester in HEPES buffer (pH 8.2, 100 mM) for at least 4 h at room temperature and 1000 rpm. Excess dye was removed by ethanol precipitation, and oligonucleotide-dye conjugates were purified by HPLC using the reverse-phase protocol above, dried under vacuum, and resuspended in nuclease-free water. Yields were determined by UV–vis measurements, and purity was confirmed by HPLC.

### Nucleic Acid Assembly Design, Fabrication, Purification

dsDNA duplexes were annealed by mixing the sense strand, modified with or without TriGalNAc, and the complementary strand, modified with AF647. Hybridization was performed in a total volume of 50 μL at a final concentration of 10 μM, by heating at 65 °C for 5 min followed by cooling from 65 to 25 °C at 1 °C per minute.

Holliday junctions were assembled as per a published protocol by mixing three LNA-modified strands, modified with or without TriGalNAc, and a final strand modified with AF647 in PBS at a final concentration of 1 μM at room temperature for 1 h and storing at 4 °C.^51^

The tetrahedron structures were folded in a one-pot self-assembly reaction by mixing AF647-modified strand 5 with the remaining strands bearing no DNA extension (TET-0×) or with DNA extensions for TET-1×, 3×, or 5× in PBS with a final concentration of 150 mM NaC. Folding occurred by incubation at 90 °C for 2 min and subsequent immediate incubation at 4 °C for minimum 10 min.

Icosahedral NANPs were designed using previously reported DNA origami frameworks for 3D wireframe structures.^66^ Custom modifications were introduced by using Tiamat to enable external overhangs for downstream functionalization. Staple sets were adapted to ensure outward-facing nicking positions for the modular assembly.

NANPs were assembled in a one-pot reaction by mixing scaffold ssDNA at a final concentration of 30 nM with a 5-fold molar excess of staple strands at a final concentration of 150 nM in 1× TAE buffer containing 12 mM MgCl_2_. Ligand–oligonucleotide conjugates were added into the folding mix with a 2.5-fold molar excess per hybridization site. Folding mixtures were incubated in a thermal cycler using the following annealing program: 65 °C for 15 min, 61 °C for 90 min, 60 °C for 90 min, and held at 25 °C. Folded NANPs were purified into 1x PBS using Amicon Ultra centrifugal filters with a MWCO of 100 kDa at 2000*g* and stored at 4 °C. For flow cytometry assays, all NANPs were fabricated with one single dye per particle, whereas NANPs for confocal microscopy were prepared with 6 dyes for improved detection.

For *in vitro* knockdown studies, purified NANPs with ssDNA overhangs were incubated with 2-fold molar excess siRNA per hybridization site at 37 °C for 10–12 h. Excess siRNA was removed using drop dialysis membranes (0.025 μm pore size) in 1× PBS at RT for 16 h. siRNA-loaded NANPs were validated for purity and monodispersity by gel electrophoresis and DLS.

### Biomolecular Corona Formation

Purified NANPs were incubated in 55% (v/v) human serum for 1 h at 37 °C to enable adsorption of serum proteins and facilitate the formation of a physiologically relevant biomolecular corona. To preserve the integrity of the formed protein corona and ensure that the corona composition remains unperturbed when modeling physiological interactions, NANPs were used directly in cell association experiments without further purification or storage.

### NANP Oligolysine_10_-PEG_5K_ Formulation

Oligolysine_10_-PEG_5K_ was formulated with NANPs as previously described.^67^ Briefly, K_10_-PEG_5k_ was dissolved in dH_2_O and sonicated at 37 °C for 10 min. 750 nM NANPs were incubated with 1000 μM oligolysine_10_-PEG_5K_ at an amino group:base pair ratio of 1 for 30 min at room temperature, before being freshly used for cell association studies.

### Agarose Gel Electrophoresis

All NANPs were analyzed by AGE using 1.6% agarose, 1× TAE buffer supplemented with 12 mM MgCl_2_, and SYBR Safe. All gels were run at 60 V for 150 min in a water bath at room temperature. Gel images were acquired on a Typhoon FLA 7000 scanner using excitation wavelengths 473 and 635 nm for SYBR safe and AF647, respectively, and processed using Fiji ImageJ software.

### Dynamic Light Scattering

Dynamic light scattering (DLS) was performed to assess the hydrodynamic diameter, dispersity, and charge of NANPs. Samples were diluted to 50 nM in 1× PBS and DLS and ζ-potentials were measured using a Malvern Zetasizer Ultra instrument; the results were analyzed using ZS XPLORER 2.0.1.1, Malvern Panalytical. LNP size, polydispersity index, and ζ-potentials were measured by using a Malvern ZetaSizer.

### Transmission Electron Microscopy

TEM grids were prepared by subjecting carbon-coated copper grids to glow discharge treatment prior to negative staining of NANPs with uranyl formate. Five μL of diluted NANPs (5 nM) was deposited onto the grid for 30 s and subsequently removed by blotting with a filter paper. Hereafter, the grids were washed with 5 μL of freshly prepared 2% uranyl formate solution with 5 mM NaOH, immediately blotted, and then washed again with 15 uL of 2% uranyl formate solution with 5 mM NaOH for 40 s. The grid was blotted and let air-dry prior to imaging on an electron microscope at 120 keV power. Images were processed in Fiji ImageJ.

### ASGPR Binding in Buffer

ICO or ICO-30T were prepared at 100 nM with 5 uM recombinant human ASGR1 in PBS, supplemented with 2 mM calcium chloride, to a total volume of 10 uL. The solution was incubated at 37 °C for 1 h and subsequently analyzed with AGE.

### Mammalian Cell Culture

HepG2, SK-HEP-1, J774A.1, and Neuro-2A cells were cultured in DMEM media supplemented with 10% FBS and 1% penicillin–streptomycin (complete medium) at 37 °C and 5% CO_2_. For plating for flow cytometry, HepG2, SK-HEP-1, and Neuro-2A cells were rinsed with PBS and released by incubation with 0.05% Trypsin-EDTA (1×) for 5–10 min at 37 °C and 5% CO_2_, whereas J774A.1 cells were released using cell scrapers. Cells were pelleted by centrifugation at 200*g* for 5 min and resuspended in complete medium. Cell count and viability were determined using Trypan Blue stain.

### Flow Cytometry

HepG2, SK-HEP-1, and J774A.1 cells were seeded (1 × 10^5^ cells/well) in a 48-well plate. After 24 h, the cells were washed with PBS and incubated with AF647-labeled NANPs at a final concentration of 50 nM in 100 μL of serum-free media for 1 h at 37 °C and 5% CO_2_. We limited the cell incubation with NANPs to 1 h because LDLR recycling reaches a steady state of receptor-bound and intracellular LDL levels within 2 h.^68,69^ Cells were then washed with PBS and detached via Trypsin-EDTA. After centrifuging the cells for 5 min at 1000*g*, the cells were washed with PBS and stained with Zombie UV Live–Dead for 10 min. After a final wash with PBS, the cells were resuspended in 150 μL of PBS and analyzed via flow cytometry. 10,000 cells were analyzed in triplicates, and after gating for live singlets, their geometric mean AF647 fluorescence intensities were calculated and normalized to untreated cells to determine relative cell association. The FlowJo software was used for the analysis of flow cytometry data.

### Antibody Blocking and Pharmacological Endocytosis Inhibition

Antihuman LDLR antibody, antihuman CD36L1 (SCARB1, SR-BI) antibody, and antihuman CD204 antibody were used to block LDL, SR-B1, and SR-A1 receptors, respectively. The cells were pretreated with 10 μg/mL antibody in 200 μL for 1 h at 4 °C followed by PBS wash and then incubated with AF647-labeled NANPs for 1 h at 37 °C and 5% CO_2_. As previously, the cells were washed, stained with Zombie UV Live–Dead stain for 10 min, and resuspended in 150 μL PBS prior to analysis on a flow cytometer.

To inhibit individual endocytic pathways, HepG2 cells were treated with 10 μM (100 μL, 1% DMSO final) Pitstop 2, dextran sulfate (40 g/mol), poly-I (40 μg/mL), methyl-β-cyclodextrin (5 mM), colchicine (25 μM), or cytochalasin D (0.25 μM). All inhibitors were incubated for 30 min except for cytochalasin D, which was incubated for 15 min at 37 °C and 5% CO_2_. The cells were then washed with PBS and incubated with AF647-labeled NANPs for 1 h under similar conditions as previously described to assay cell association using flow cytometry.

### Confocal Microscopy

HepG2 cells were seeded (80,000 cells/well) in 12 well chamber removable microscopy slides. After 24 h, the cells were rinsed with PBS and incubated with 100 nM NANP preincubated with 55% human serum in a final volume of 100 uL serum-free DMEM media for 24 h at 37 °C and 5% CO_2_. The following day, the cells were washed with PBS three times and then stained with Wheat Germ Agglutinin (WGA) Alexa Fluor 488 (2.5 μg/mL) for 15 min at 37 °C and 5% CO_2_. Subsequently, the cells were rinsed with PBS twice and fixed in 4% paraformaldehyde (PFA) for 20 min at room temperature. Following PBS washes, the cells were stained with DAPI nucleic acid stain (1 μg/mL) for 20 min at room temperature. Following two PBS washes, the chambers were removed and the slide was rinsed with Milli-Q water, dried, and mounted using ProLong Gold Antifade mounting medium overnight prior to confocal imaging using 63× objectives. All images were acquired with identical laser and filter settings and subsequently visualized as maximum intensity Z-projections with brightness and contrast equally adjusted in Fiji ImageJ. Orthogonal XY/XZ views were generated from the corresponding raw Z-stacks for the ICO-6C particle.

### Enzyme-Linked Immunosorbent Assay (ELISA) for the Quantification of Apolipoprotein

To assess apolipoprotein recruitment to DNA nanoparticles, high-binding 96-well Nunc Maxisorp plates were coated with 100 μL of 10 μg/mL poly-d-lysine at 37 °C for 30 min. The plates were washed once with phosphate-buffered saline (PBS), and 50 μL of NANP solution (20 μg/mL in PBS) was added to each well and incubated overnight at 4 °C. The following day, the plates were washed once with PBS and blocked with 50 μL of blocking buffer, constituting PBS containing calcium and magnesium supplemented with 1% bovine serum albumin (BSA) for 2 h at room temperature. The plates were then washed four times with PBS containing 0.05% Tween-20 (PBS-T), with blotting between each wash.

To simulate *in vivo* conditions and facilitate biomolecular corona formation, plated NANPs were incubated in 55% human serum diluted in blocking buffer (100 μL/well) for 1 h at 37 °C. After four PBS-T washes, the plates were incubated with 50 μL of 10 μg/mL mouse antihuman apolipoprotein B (ApoB) or apolipoprotein E (ApoE) antibodies diluted in blocking buffer for 1 h at room temperature. The plates were washed again four times with PBS-T, followed by a 1 h incubation with 50 μL of horseradish peroxidase (HRP)-conjugated goat antimouse IgG secondary antibody diluted 1:5000 in blocking buffer. After a final set of four PBS-T washes, 50 μL of 1-Step Ultra TMB-ELISA substrate was added to each well and developed for 5 min in the dark. The enzymatic reaction was quenched with 25 μL of 2 M sulfuric acid, and absorbance was measured at 450 nm with a reference wavelength of 540 nm.

Standard curves for ApoB and ApoE were generated by plating serial dilutions of each protein and measuring the absorbance. Data were fit to a four-parameter logistic model to establish the relationship between concentration and absorbance and enable the interpolation of protein concentrations from sample absorbance values.

### LNP-siRNA Formulation

Nanoparticle formulations were done as previously described.^70^ Briefly, DLin-MC3-DMA (MC3), 1,2-distearoyl-*sn*-glycero-3-phosphocholine (DSPC), cholesterol, and DMG-PEG2000 were mixed in ethanol at a molar ratio of 50:10:38.5:1.5 as the organic phase. RNA was prepared in 10 mM citrate buffer, pH 3, at an RNA concentration such that the final RNA concentration is 0.1 μg/μL following microfluidic mixing. The formulated aqueous and organic phases were mixed at a ratio of 3:1, respectively, using a syringe pump and microfluidic chip setup as previously described. Subsequently, nanoparticles were buffer-exchanged into PBS by several rounds of centrifugation using Amicon centrifugal filters with MWCO = 100 kDa. Encapsulation efficiency and RNA concentrations were measured using Quant-iT Ribogreen assay as previously described.^70^ Briefly, RNA concentrations were determined in Tris-EDTA buffer (TE) and Tris-EDTA, 1% Triton-X 100 buffer (TX). Encapsulation efficiency (EE) was determined by using the following formula: EE = (TX – TE)/TX × 100%.

### *In Vitro* RNAi Experiments

HepG2 cells were seeded onto a 96-well plate (1 × 10^4^ cells/well). After 24 h, the cells were washed with PBS and incubated with siRNA and/or NANP in a final volume of 100 μL of serum-free media. Media was exchanged 24 h later to complete DMEM. Total RNA was isolated using 50 μL of iScript RT-qPCR Sample Preparation Reagent per well. 10 μL was then transferred to generate cDNA using the High-Capacity cDNA Reverse Transcription Kit. ALDH2 expression was analyzed via RT-qPCR using Taqman probes for ALDH2 (FAM) and HPRT (VIC) primers in a dual-probe assay. The delta–delta Ct method was used to analyze Ct values and compute the fold reduction in ALDH2 expression.

### *In Vivo* RNAi Experiments

Female C57BL/6 mice (strain no. 000664) were purchased from The Jackson Laboratory (Bar Harbor, ME). 200 μL of either PBS, bare siRNA, or TriGalNAc-siRNA diluted in PBS were administered intravenously via the tail vein. 72 h later, mice were euthanized by CO_2_ asphyxiation and cervical dislocation and the livers were harvested and flash-frozen in liquid nitrogen. Frozen livers were homogenized to dry powder using a Genogrinder TissueLyzer. 5 mg of liver tissue was collected into an Eppendorf tube for subsequent RNA isolation using the Qiagen RNEasy Mini (Plus) kit following the manufacturer’s guidelines with some modifications. Briefly, 350 μL of RLT buffer supplemented with β-mercaptoethanol was added to the tissues and vortexed rigorously before adding 70% ethanol. The lysate was transferred to a RNeasy spin column and centrifuged at 8000*g* for 30 s. 700 μL of RW1 buffer was added to the spin columns and centrifuged for 30 s following addition of 500 μL of RPE buffer. The latter step was repeated in total thrice; the column was then centrifuged for 1 min to dry followed by elution of the RNA with 30 μL of nuclease-free water. 10 μL of purified RNA was used for subsequent cDNA synthesis and qPCR analysis.

### Statistical Analysis

All statistical analyses were conducted in GraphPad Prism v.10. All data are presented as the mean ± standard deviation. *P*-values are indicated in the individual figure panels. The specific statistical method used and the number of replicates are described in the figure legends.

## Supplementary Material





## Notes

Raw data (XLSX)
Lipophilicity calculation; additional NANP and LNP characterization; additional flow cytometry and cell association characterization; additional confocal microscopy characterization; additional ELISA characterization; RNAi assessment; and sequence information (PDF)
