# Supplementary material for: Programmable Lipid Functionalization of Nucleic Acid Nanoparticles Modulates Liver Cell-Type Targeting
Source: ACS Appl Mater Interfaces. 2026 Mar 25;18(13):18721–39. doi: 10.1021/acsami.5c24581 (PMC13067242; doi:10.1021/acsami.5c24581)
Supplement: Supplementary file 2 [file am5c24581_si_002.pdf]

Supporting Information

Title

Programmable Lipid Functionalization of Nucleic Acid Nanoparticles Modulates Liver Cell-type Targeting

Authors

Hyun Min Kim[1], Marjan Omer[1,2], Grant A. Knappe[1,3], Patrick McMullen[4, 5], Duy An Le[1], Ashwin Pasupathy[3,4], Daniel G. Anderson[3,4,5,6,7], Mark Bathe[1,8,9,*]

Affiliations

[1]Department of Biological Engineering, Massachusetts Institute of Technology, Cambridge, Massachusetts, 02139, United States

[2]Department of Biomedicine, Aarhus University, 8000 Aarhus C, Denmark

[3]Department of Chemical Engineering, Massachusetts Institute of Technology, Cambridge, Massachusetts, 02139, United States

[4]David H Koch Institute for Integrative Cancer Research, Massachusetts Institute of Technology, Cambridge, Massachusetts, 02139, United States

[5]Department of Anesthesiology, Boston Children's Hospital, Boston, Massachusetts, 02115, United States

[6]Harvard and MIT Division of Health Science and Technology, Massachusetts Institute of Technology, Cambridge, Massachusetts, 02139, United States

[7]Institute for Medical Engineering and Science, Massachusetts Institute of Technology, Cambridge, Massachusetts, 02139, United States

[8]Broad Institute of MIT and Harvard, Cambridge, Massachusetts, 02142, United States

[9]Harvard Medical School Initiative for RNA Medicine, Harvard Medical School, Boston, Massachusetts, 02115, United States

*Correspondence to: mark.bathe@mit.edu

Supporting Notes

Note S1. Lipophilicity calculations of lipids used in this study.

To approximate the lipophilicity of the different lipids used in this study, we calculated the partition coefficient between n-octanol and water (logP), generally used as a measure of a molecules logP. The logP for the cholesterol, C18, and palmitate ligands were 10.02, 8.01, and 7.43, respectively (ChemDraw 20.1.0.110).

Supporting Figures

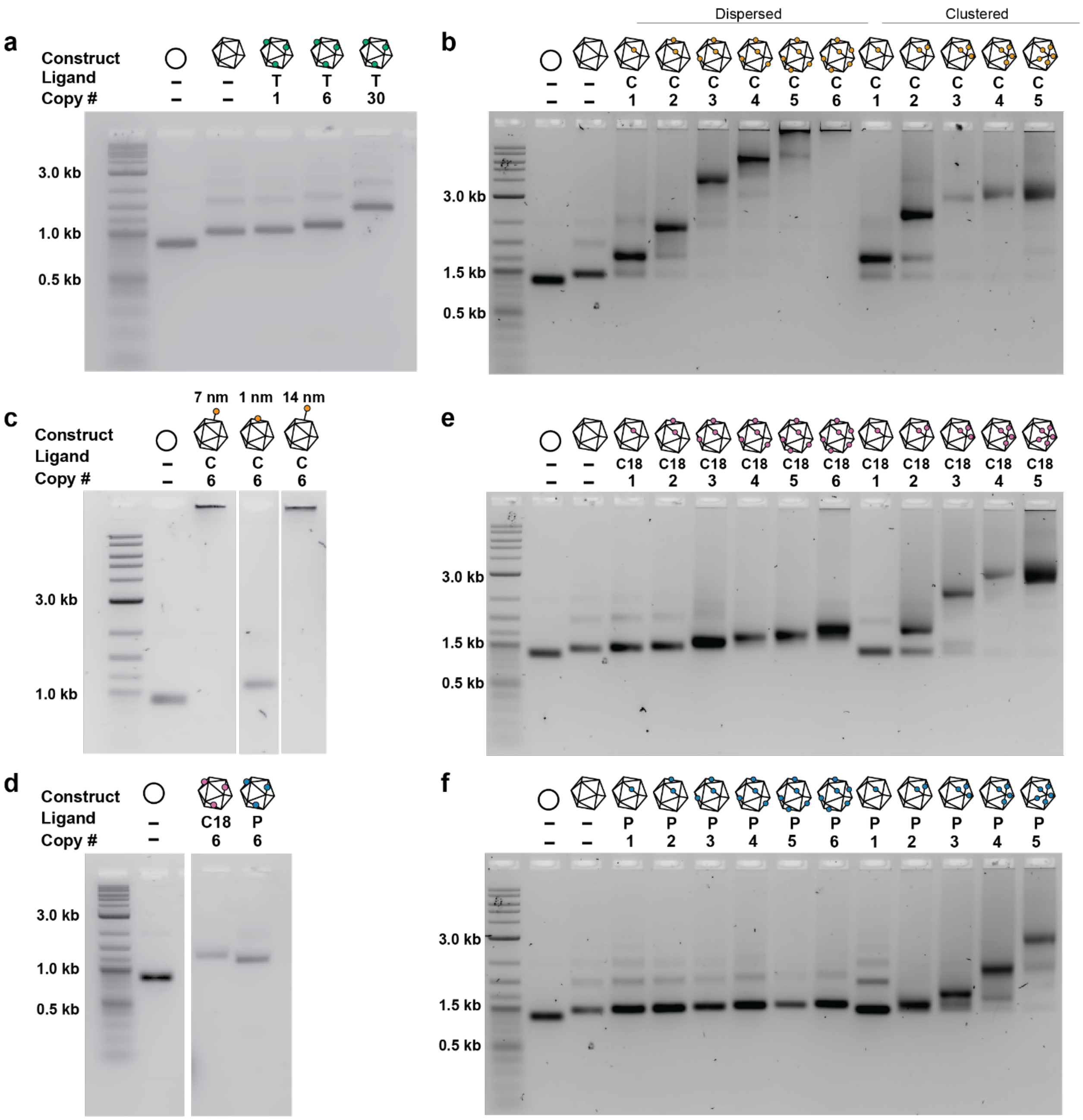

Figure S1. Characterization of ligand-modified nanoparticles used in this study. a AGE gel characterization of 3120 nt scaffold, bare ICO and ICO with 1, 6 or 30 copies of TriGalNAc (T). **b** AGE gel analysis of purified ICO NANPs with 1-6 copies of dispersed cholesterol (C) or 1-5 copies of clustered cholesterol. **c** AGE gel characterization of ICO-6C with varying linker length (from left 7 nm, 1 nm and 14 nm). **d** AGE gel analysis of 3120 nt scaffold, ICO with 6 copies of C18 or Palmitate (P) with a linker length of 7 nm. **e** AGE gel analysis of 3120 nt scaffold, bare ICO or ICO NANPs modified with varying number of C18 in dispersed or clustered configurations similar as (b). **f** AGE gel analysis of 3120 nt scaffold, bare ICO, or ICO conjugated with 1-6 copies of Palmitate (P) in dispersed or clustered configurations.

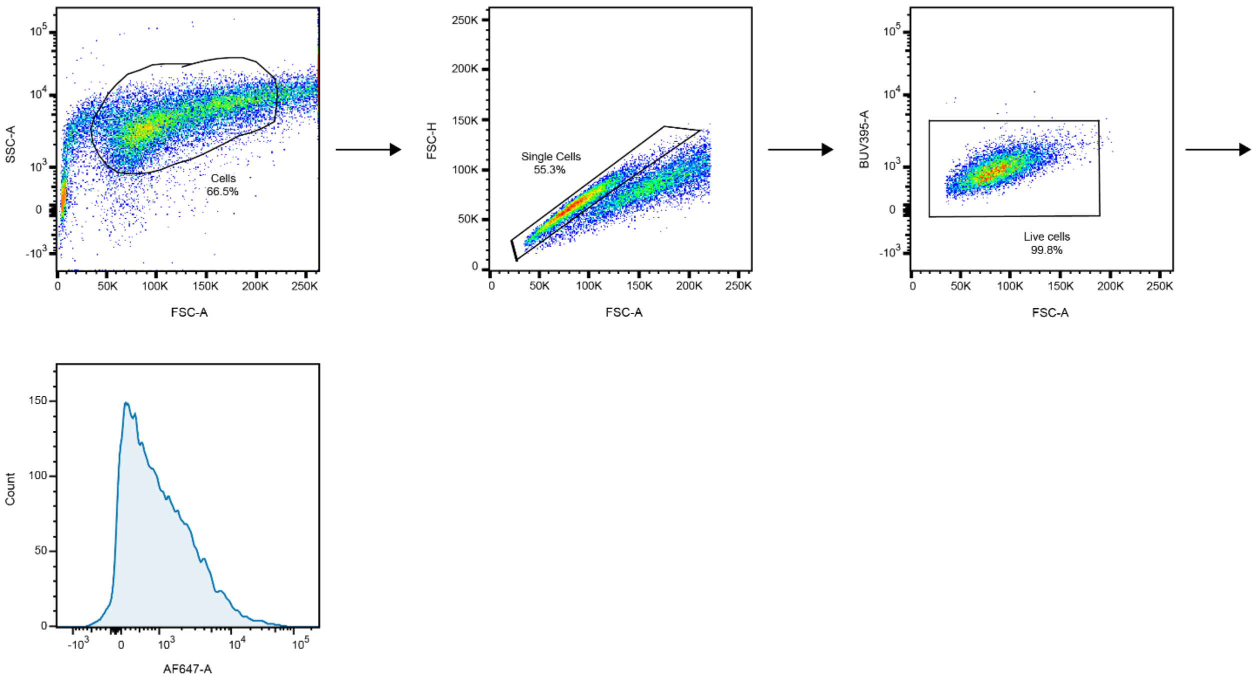

Figure S2. Gating strategy for flow cytometry analysis in this study. Representative gating strategy used for cell association studies in HepG2, SK-HEP-1 and J774A.1 cells in this study. Live cells were gated to identify AF647 signal under different conditions.

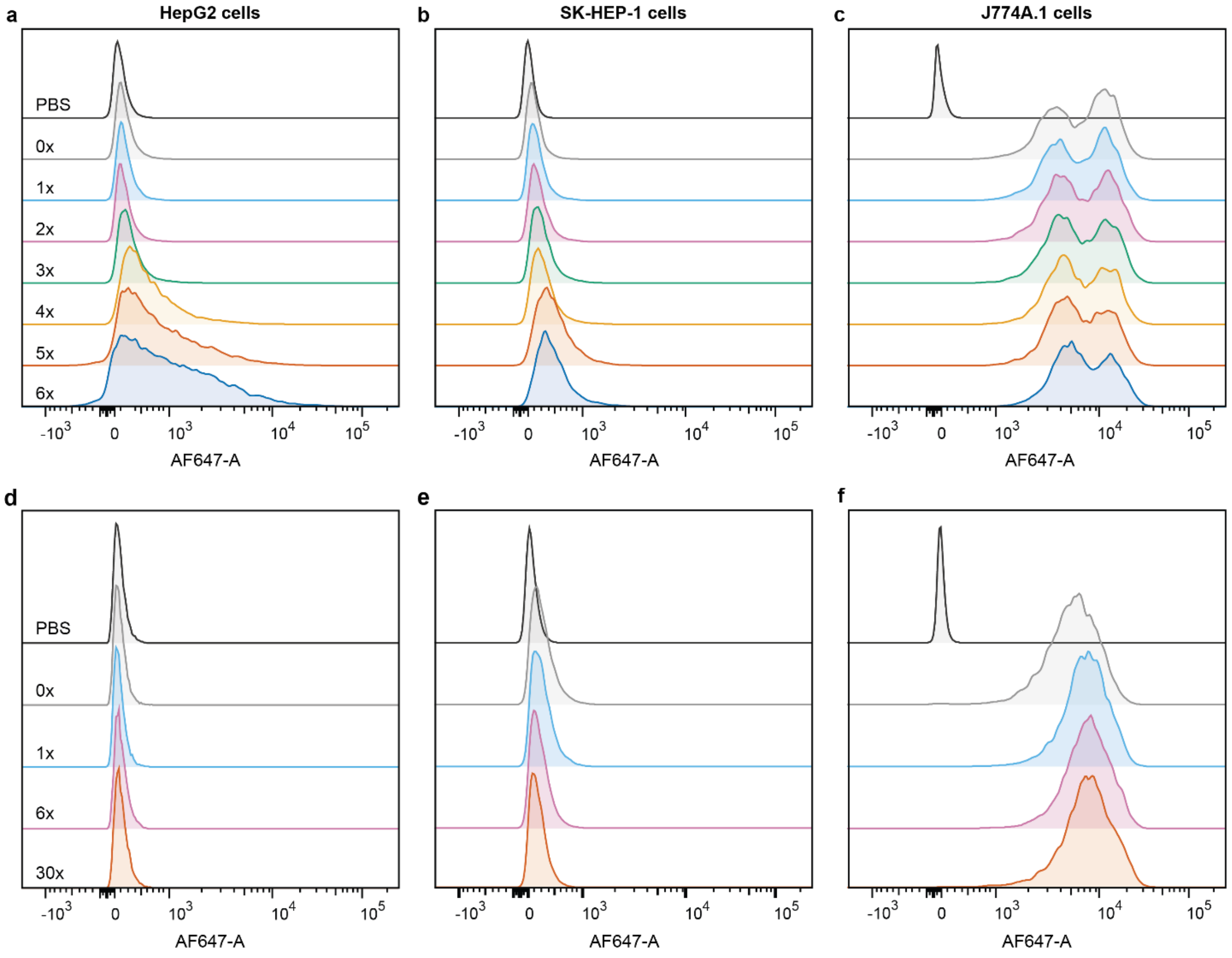

Figure S3. Representative flow cytometry histograms of NANP cell association in HepG2, SK-HEP-1 and J774A.1 cells. Cells were incubated for 1 hour with PBS or 50 nM AF647-labeled ICO-*n*C NANPs carrying 0-6 copies cholesterol and preincubated with 55% human serum (top row, panels **a-c**) or 50 nM AF647-labeled ICO-*n*T NANPs bearing 0-30 TriGalNAc ligands (bottom row, panels **d-f**).

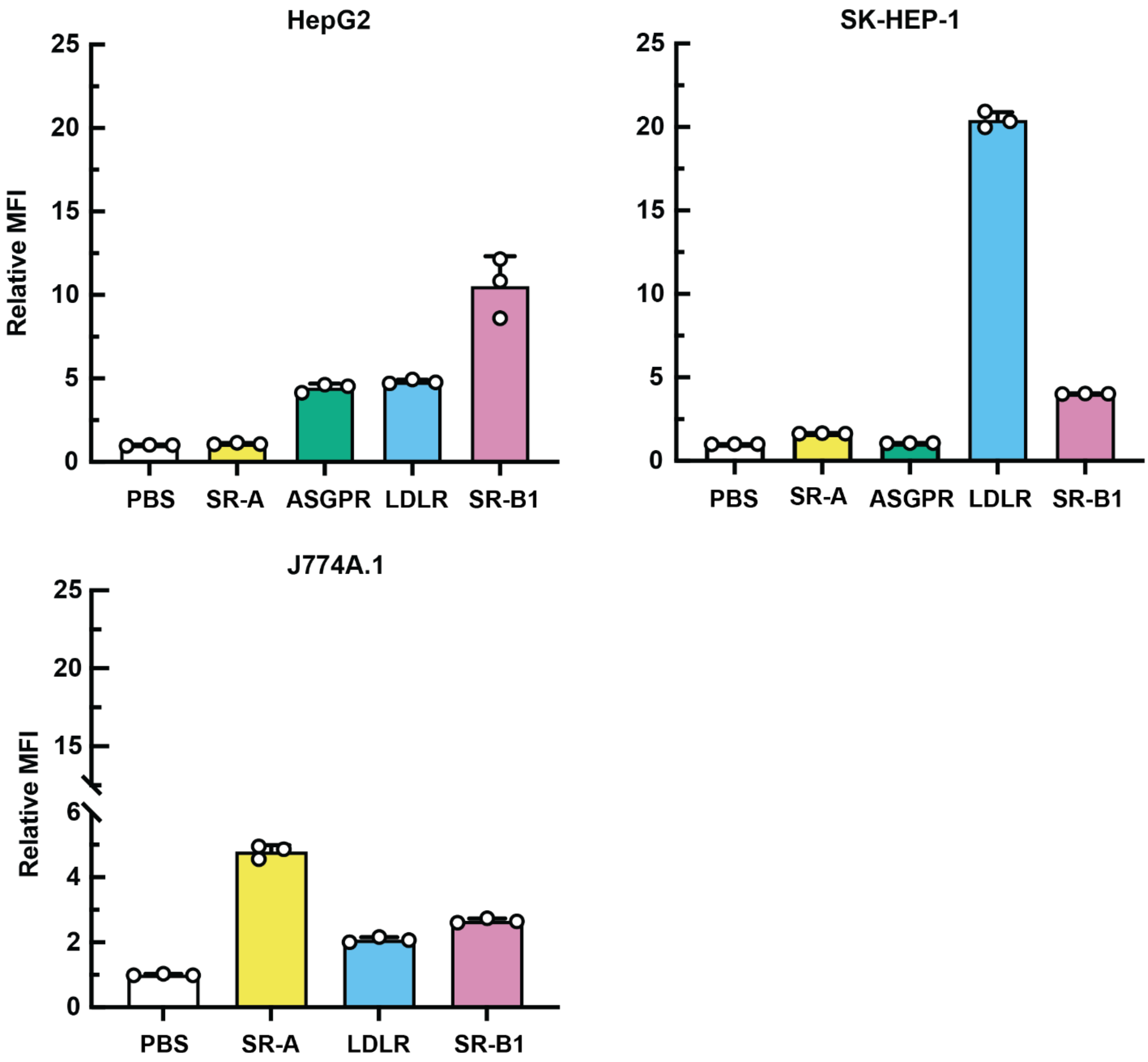

Figure S4. Receptor expression profiles in liver cell types. AF647-labeled antibodies were used to determine the receptor expression profiles in the cell lines HepG2 (left), SK-HEP-1 (right), and J774A.1 (bottom) by flow cytometry. Error bars indicate mean ± SD, n=3 biological replicates. SRA: scavenger receptor class A; ASGPR: asialoglycoprotein receptor; LDLR: Low density lipoprotein receptor; SRB1: scavenger receptor class B type I. MFI: mean fluorescence intensity.

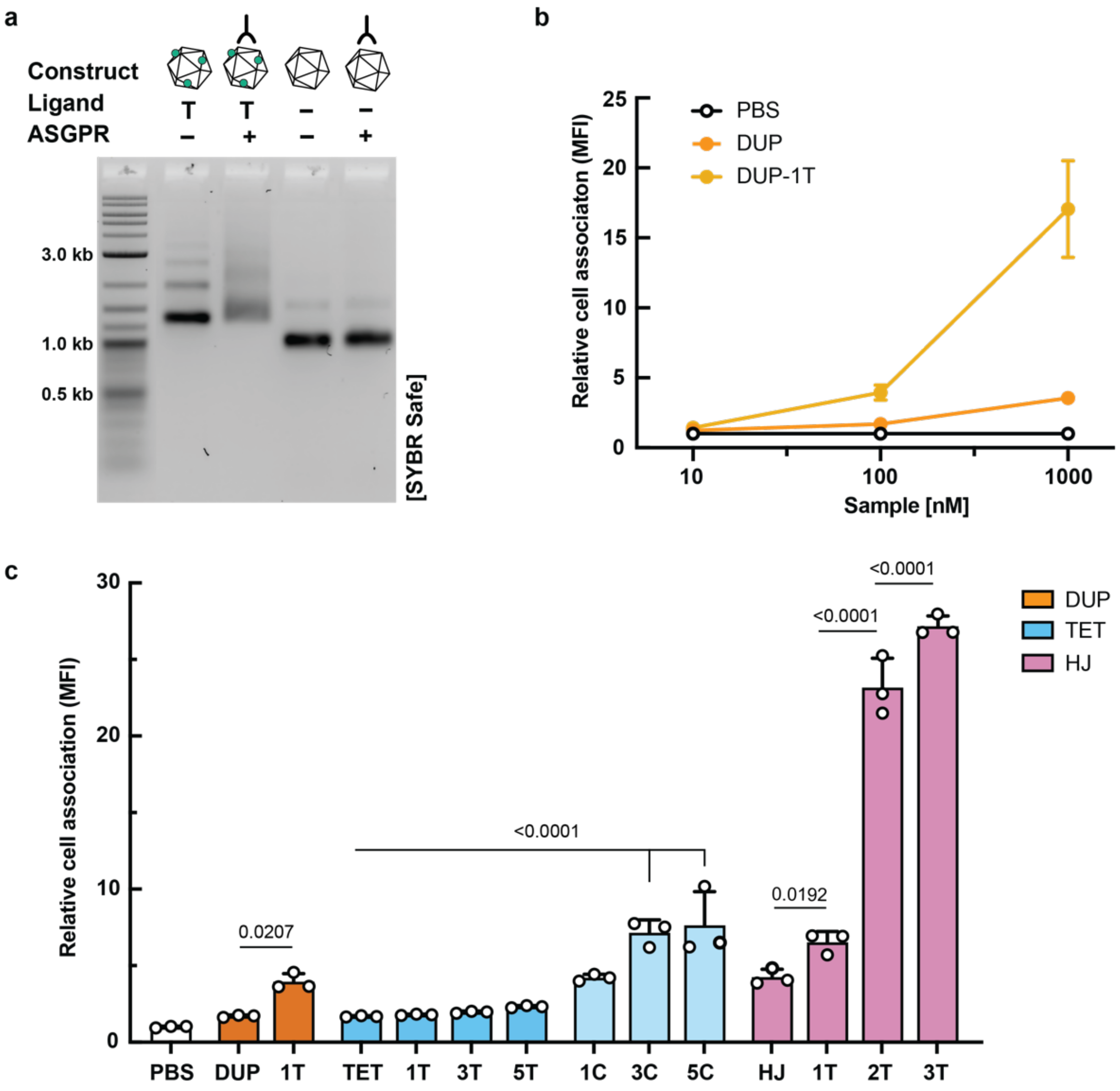

Figure S5. TriGalNAc-mediated receptor cell association of DNA nanostructures of varying size and complexity. a AF647-labeled bare ICO or TriGalNAc-modified ICO was incubated with soluble ASGPR protein and analyzed by 1.6% AGE gel. A modest gel shift was observed in lane 3 (from left) indicating that ICO-TriGalNAc binds to its cognate receptor, ASGPR, whereas no shift is observed for the bare ICO. **b** Cell association of duplex DNA (DUP) or duplex DNA with one TriGalNAc (DUP-1T) at varying concentrations in HepG2 cells following 1 hour incubation. **c** Relative cell association in HepG2 cells following 1 hour incubation with 50 nM of AF647-labeled constructs: DNA duplex, tetrahedral (TET)[1] and Holliday Junction-like structures[2], with an increasing copy number of TriGalNAc (T) or cholesterol (C) (only for tetrahedral structure). Error bars indicate mean ± SD, n=3 biological replicates. Statistical analysis was conducted by One-way ANOVA using Tukey's post hoc test for multiple comparisons; p-values are indicated. MFI: mean fluorescence intensity.

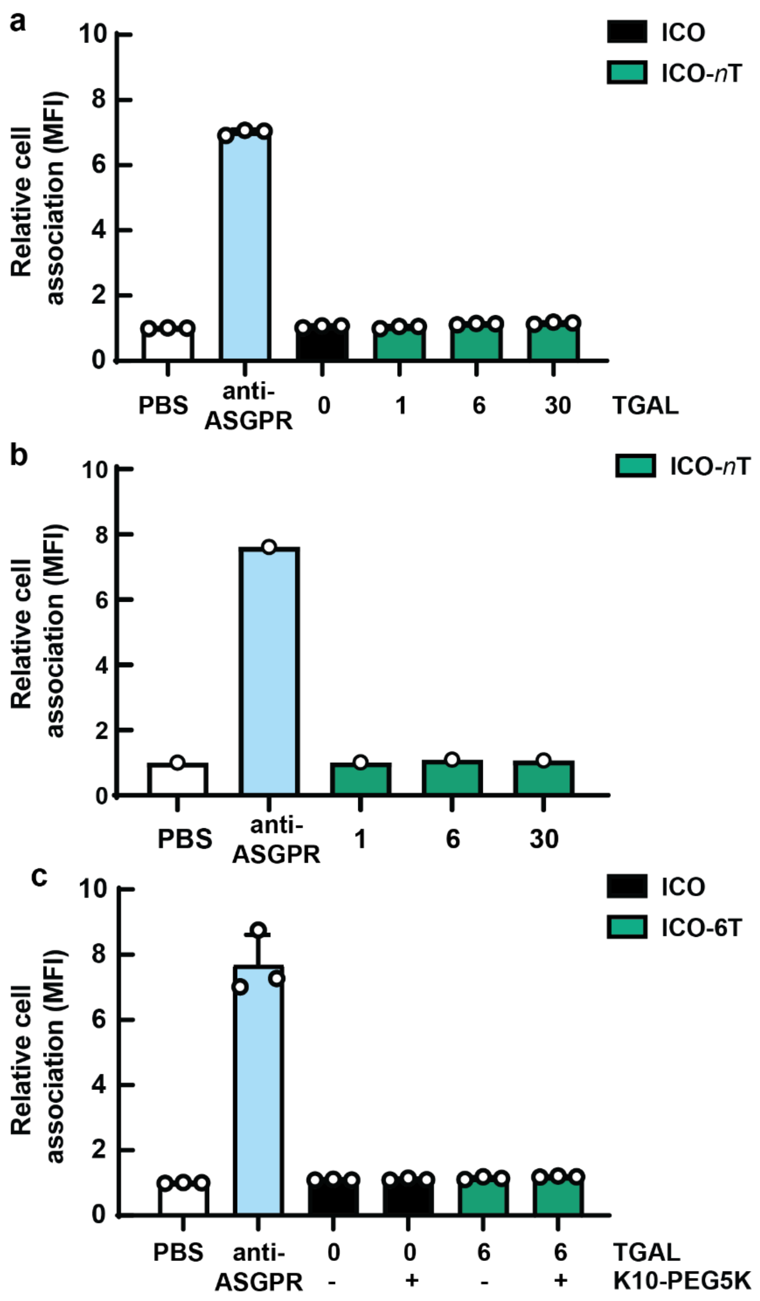

Figure S6. Assessment of serum effects, linker length, and surface-charge modulation on TriGalNAc-mediated NANP association with HepG2 cells. a Relative HepG2 cell association following 1 hour incubation of 50 nM bare ICO or ICO with 1, 6 or 30 copies of TriGalNAc (T) in absence of serum. **b** Relative HepG2 cell association of 50 nM ICO-*n*T using a 40mer dsDNA linker to present TriGalNAc ligands on NANP. Greater ligand accessibility did not improve cell association. **c** HepG2 cells were treated with 100 nM of bare ICO with or without oligolysine-PEG5K (K10-PEG5K), or ICO-6T with or without K10-PEG5K for 1 hour prior to flow cytometry analysis. Error bars indicate mean ± SD, n=3 biological replicates. Anti-human ASGPR antibody was used as positive control. ICO and ICO-6T formulated with K10-PEG5k had a zeta potential of -3.3 and -1.9 mV, respectively (N=1 biological replicate). Error bars represent mean ± SD, n=3 biological replicates.

a b

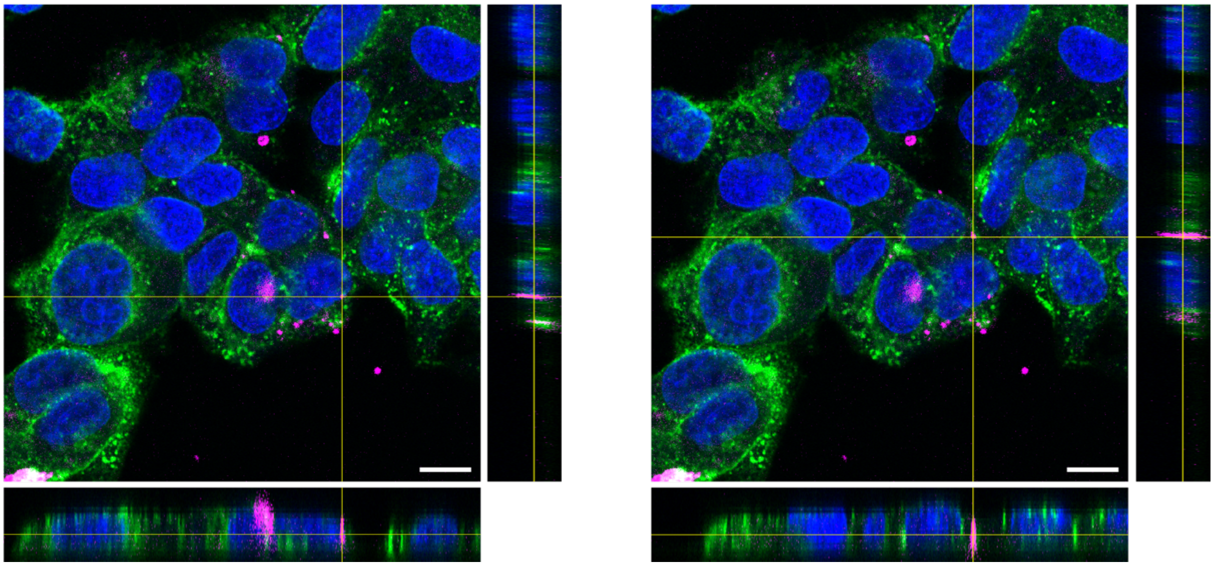

Figure S7. Supporting information for confocal microscopy of ICO-6C in HepG2 cells. Cells were treated with 100 nM AF647-labeled ICO-6C for 24 hours. Two representative orthogonal views of Z-stack projection show **a** internalized ICO-6C and **b** cell surface-associated ICO-6C. Magenta: AF647-labeled ICO-6C; blue: DAPI nucleus stain; and green: WGA membrane stain. Scale bar: 10 μm.

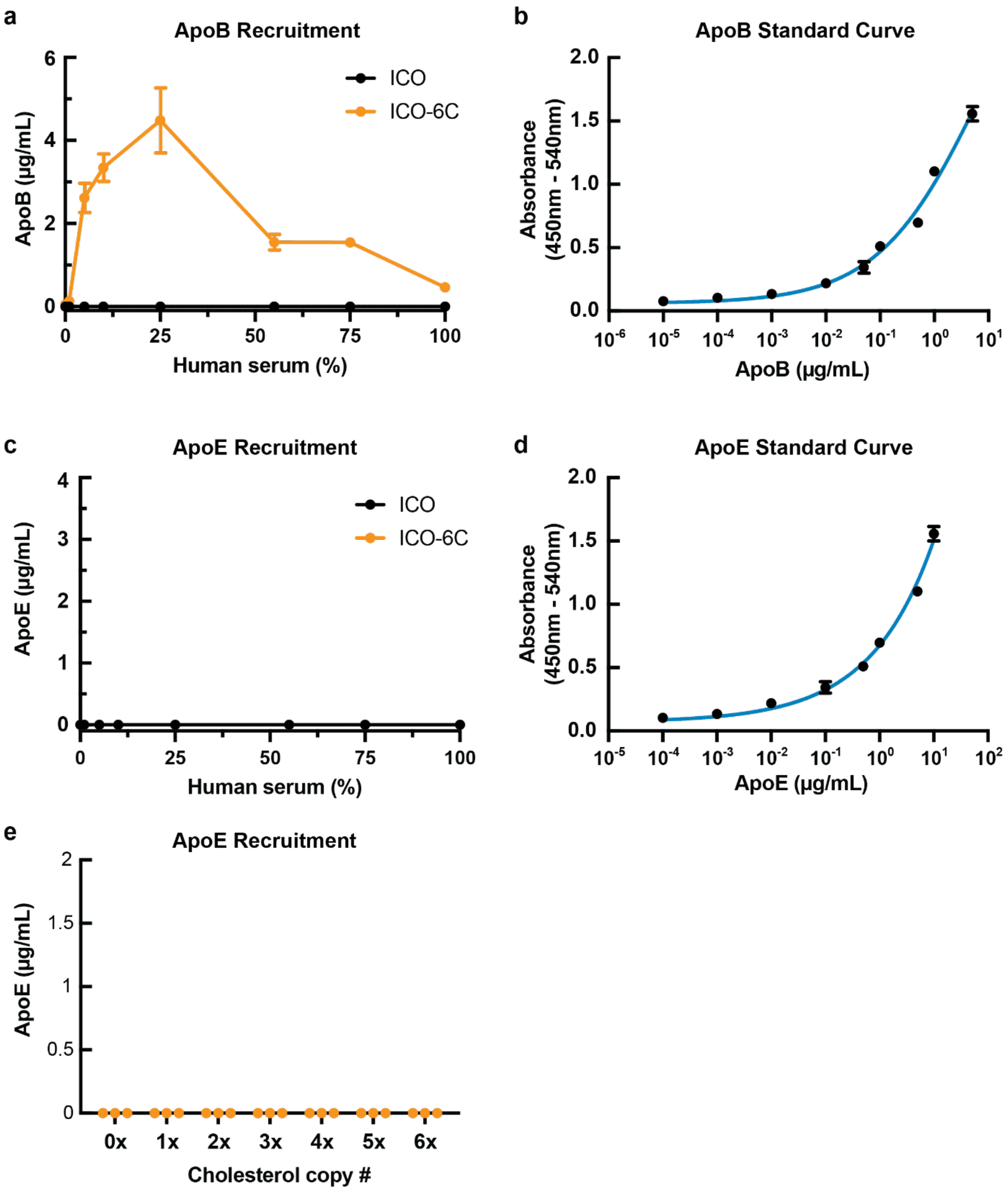

Figure S8. Evaluation of ApoB and ApoE in biomolecular corona by ELISA assay. ELISA assays were conducted to assess the recruitment of ApoB (**a**) and ApoE (**c**) mediated by ICO or ICO-6C NANPs under varying human serum concentrations. ELISA plates were coated with NANPs prior to incubation with human serum for 1 hour at 37 °C prior to ELISA. Protein concentrations were quantified based on standard curves for ApoB (**b**) and ApoE (**d**) using soluble proteins. **e** ApoE ELISA using ICO NANPs with varying cholesterol copies did not lead to ApoE recruitment.

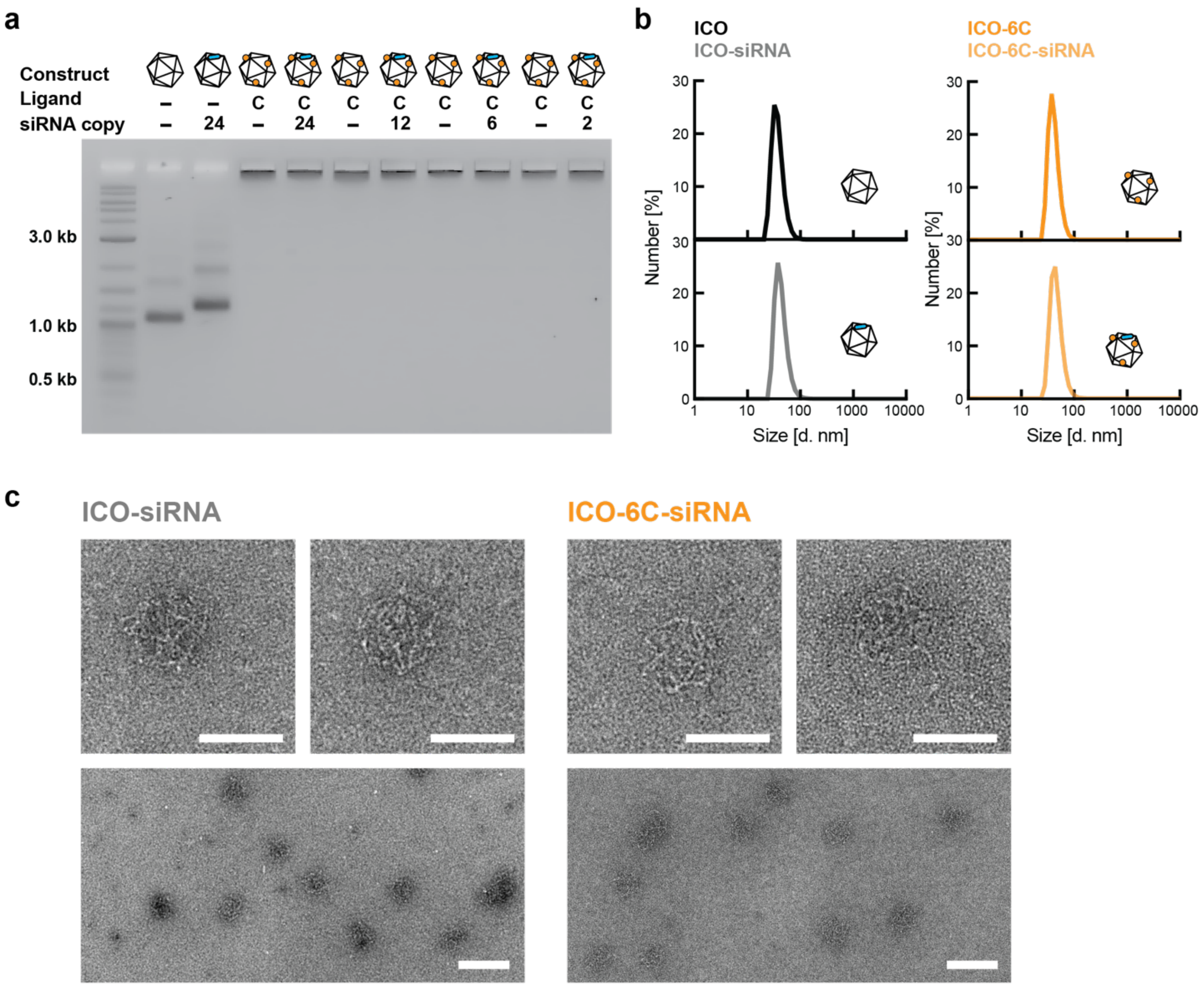

Figure S9. Characterization of NANPs with siRNA and cholesterol functionalization. a Gel characterization of bare ICO, ICO with 24 siRNAs, and cholesterol-modified ICO with varying copy number of siRNA. **b** Size distribution of ICO with barcodes (BC1) with and without siRNA (ICO and ICO-siRNA) and ICO-6C modified with 24 BC1 with and without siRNA (ICO-6C-siRNA). **c** TEM electron micrographs of ICO- and ICO-6C with 24 siRNA structures. Scale bars 50 nm (top row) and widefield image (bottom row) 100 nm.

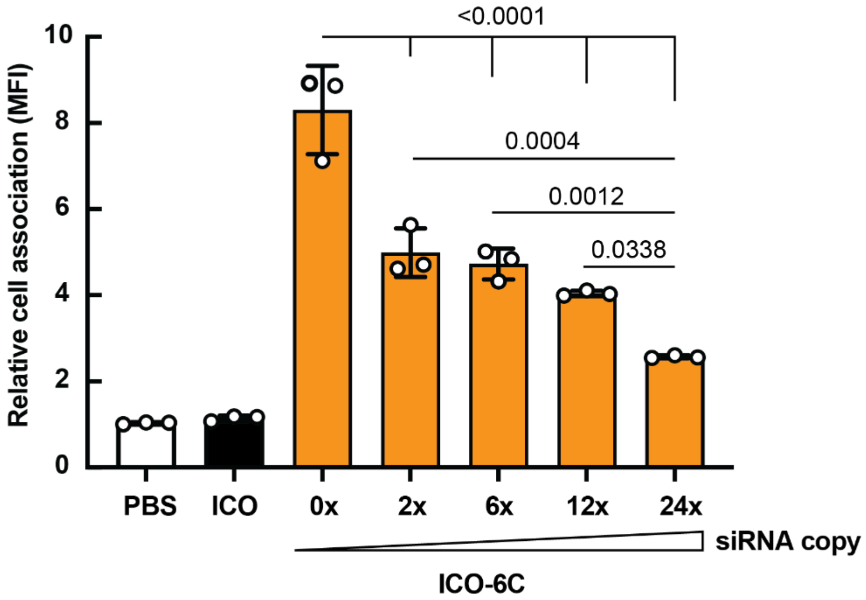

Figure S10. Effect of siRNA valency on cell association of cholesterol-modified NANPs. Relative cell association in HepG2 cells incubated with 50 nM AF647-labeled bare ICO or ICO-6C with an increasing siRNA copy number (2, 6, 12 and 24) for 1 hour prior to flow cytometry analysis. The mean fluorescence intensity (MFI) of samples was normalized to PBS control. Error bars indicate mean ± SD, n=3 biological replicates. Statistical analysis was conducted by One-way ANOVA using Tukey's post hoc test for multiple comparisons; p-values are indicated.

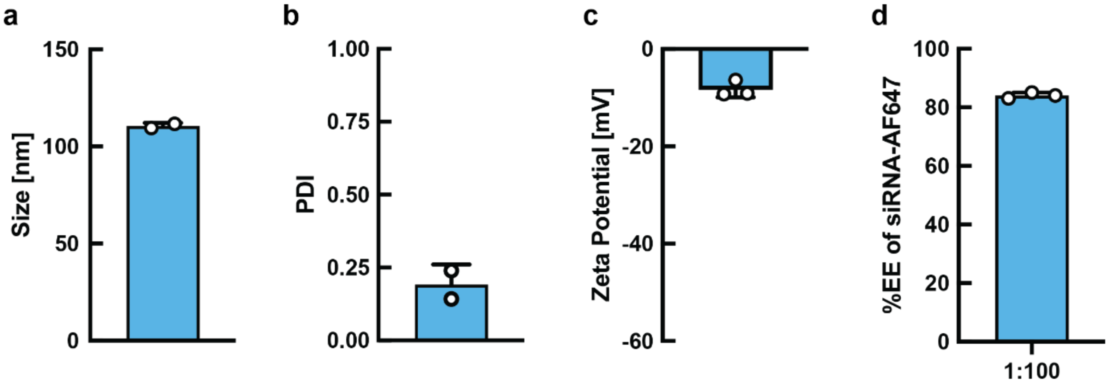

Figure S11. siRNA encapsulated LNP characterization. a DLS size (diameter) analysis of LNP-siRNA formulation. **b** Polydispersity index (PDI). **c** Zeta potential measurement of LNP-siRNA. d siRNA-AF647 encapsulation efficiency quantified by Quant-iT Ribogreen assay at 1:100 dilution using the formula given in Methods section. Error bars indicate mean ± SD.

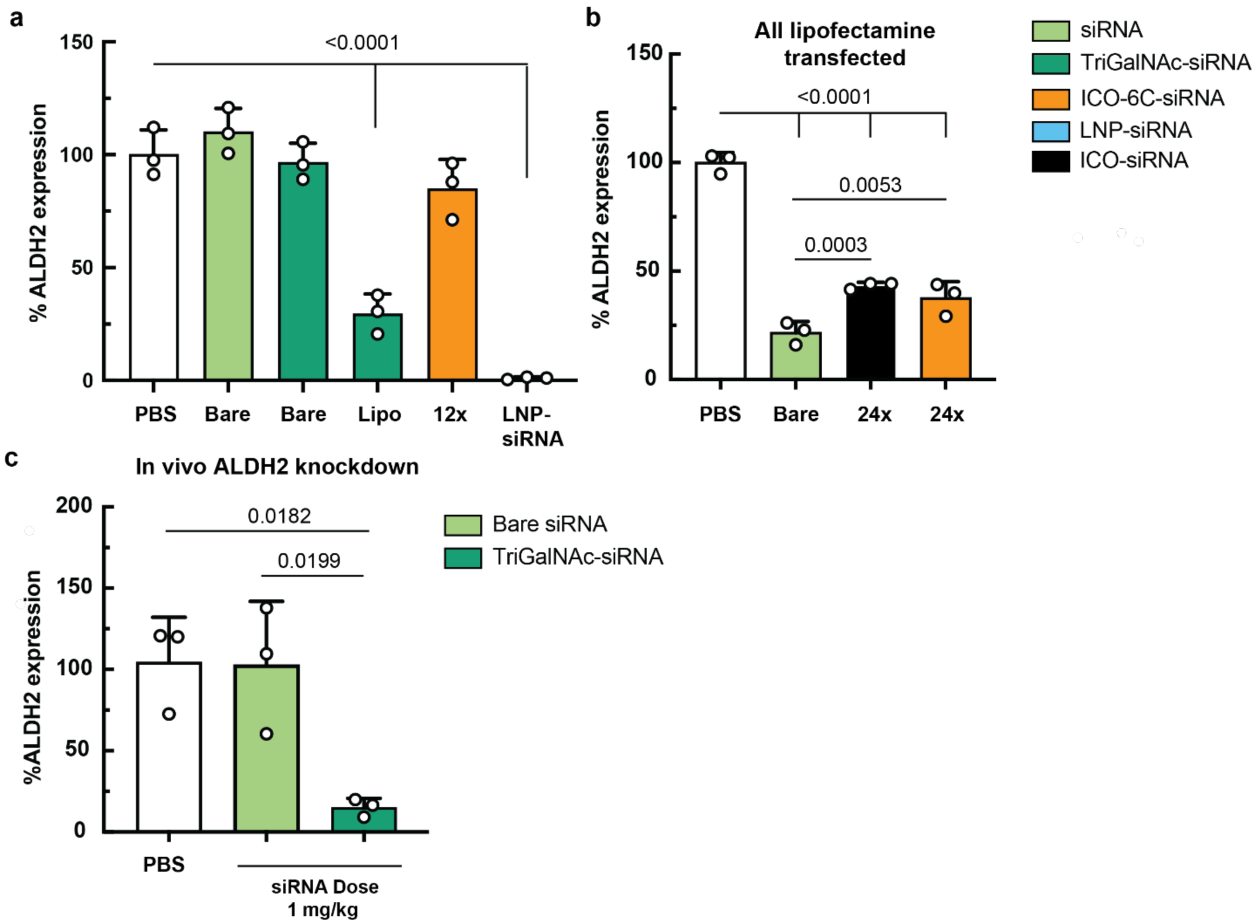

Figure S12. Evaluation of ALDH2 knockdown by siRNA and NANP delivery in HepG2 cells and C57BL/6 mice. a Knockdown of ALDH2 in HepG2 cells following autonomous or lipofectamine-mediated transfection with bare siRNA, ICO or ICO-6C modified with 24 siRNAs at a final siRNA concentration of 25 nM. **b** ALDH2 knockdown after incubation with PBS, bare siRNA, TriGalNAc-siRNA, lipofectamine-complexed TriGalNAc-siRNA, ICO-6C bearing 12 siRNAs, or LNP encapsulated siRNA (LNP-siRNA) at final siRNA concentration of 10 nM. **c** Knockdown of ALDH2 gene in C57BL/6 mice following intravenous administration of PBS, bare siRNA or TriGalNAc-siRNA at 1 mg/kg. These results confirm that the TriGalNAc-siRNA works in mouse models, but not in the in vitro HepG2 hepatocyte cell line. Error bars indicate mean ± SD, n=3 biological replicates. Statistical analysis was conducted by One-way ANOVA using Tukey's post hoc test for multiple comparisons; p-values are indicated.

Supporting Tables

Table S1. ICO52 scaffold sequence (3120 nt)

GAGCGCAACGCAATTAATGTGCGCCCTGTAGCGGCGCATTAAGCGCGGCGGGTGTGGTGGTTACGCGCAGCG
TGACCGCTACACTTGCCAGCGCCCTAGCGCCCGCTCCTTTCGCTTTCTTCCCTTCCTTTCTCGCCACGTTCGCC
GGCTTTCCCCGTCAAGCTCTAAATCGGGGGCTCCCTTTAGGGTTCCGATTTAGTGCTTTACGGCACCTCGACCC
CAAAAAACTTGATTAGGGTGATGGTTCACGTAGTGGGCCATCGCCCTGATAGACGGTTTTTCGCCCTTTGACGTT
GGAGTCCACGTTCTTTAATAGTGGACTCTTGTTCCAAACTGGAACAACACTCAACCCTATCTCGGTCTATTCTTTT
GATTTATAAGGGATTTTGCCGATTTCGGCCTATTGGTTAAAAAATGAGCTGATTTAACAAAAATTTAACGCGAATT
ACAACCGGGGTACATATGATTGGGGTCTGACGCTCAGTGGAACGAAAACTCACGTTAAGGGATTTTGGTCATGA
GATTATCAAAAAGGATCTTCACCTAGATCCTTTTAAATTAAAAATGAAGTTTTAAATCAATCTAAAGTATATATGAG
TAAACTTGGTCTGACAGTTACCAATGCTTAATCAGTGAGGCACCTATCTCAGCGATCTGTCTATTTCGTTCATCCA
TAGTTGCCTGACTCCCCGTCGTGTAGATAACTACGATACGGGAGGGCTTACCATCTGGCCCCAGTGCTGCAATG
ATACCGCGAGACCCACGCTCACCGGCTCCAGATTTATCAGCAATAAACCAGCCAGCCGGAAGGGCCGAGCGCA
GAAGTGGTCCTGCAACTTTATCCGCCTCCATCCAGTCTATTAATTGTTGCCGGGAAGCTAGAGTAAGTAGTTCGC
CAGTTAATAGTTTGCGCAACGTTGTTGCCATTGCTACAGGCATCGTGGTGTCACGCTCGTCGTTTGGTATGGCTT
CATTCAGCTCCGGTTCCCAACGATCAAGGCGAGTTACATGATCCCCCATGTTGTGCAAAAAAGCGGTTAGCTCC
TTCGGTCCTCCGATCGTTGTCAGAAGTAAGTTGGCCGCAGTGTTATCACTCATGGTTATGGCAGCACTGCATAAT
TCTCTTACTGTCATGCCATCCGTAAGATGCTTTTCTGTGACTGGTGAGTACTCAACCAAGTCATTCTGAGAATAGT
GTATGCGGCGACCGAGTTGCTCTTGCCCGGCGTCAATACGGGATAATACCGCGCCACATAGCAGAACTTTAAAA
GTGCTCATCATTGGAAAACGTTCTTCGGGGCGAAAACTCTCAAGGATCTTACCGCTGTTGAGATCCAGTTCGATG
TAACCCACTCGTGCACCCAACTGATCTTCAGCATCTTTTACTTTCACCAGCGTTTCTGGGTGAGCAAAAACAGGA
AGGCAAAATGCCGCAAAAAAGGGAATAAGGGCGACACGGAAATGTTGAATACTCATACTCTTCCTTTTTCAATAT
TATTGAAGCATTTATCAGGGTTATTGTCTCATGAGCGGATACATATTTGAATGTATTTAGAAAAATAAACAAATAGG
GGTTCCGCGCACATTTCCCCGAAAAGTGCCACCTGACGTCTAAGAAACCATTATTATCATGACATTAACCTATAA
AAATAGGCGTATCACGAGGCCCTTTCGTCGAATTCGTCGTCGTCCCCTCAAACTCTTGGGTGGAGAGGCTATTC
GTTTAAGGTCACATCGCATGTAATTTACTTATTCTCTGTTGTTGAGCCACCCGGGCGCCAGATTTTGTTTAAAGCT
TTGTCTCTTAGTTTGTATAGACAGATTCAGAGTGCAAGGTTTCGTTCGCTCGTACCTGGTTTTCCCTGGTTCTTCA
CAGATAGGATTTGACTTTCTACAACACTTATGCGGCTTCCTACCCGTTTGAAGGCCGATACAGGTGCTGCGCAAA
ATGCGGGCGAACATAGAGTATCAAAACAACGCCTTCTAATCTAGGAATATAGGGAAGATACGTATTTGCTACCAT
GCTTTCTTGGGTCATTAACGACCAACCTCTTTTCTTTTAAAGTAGGATTGCACAATGAATGAATACACGTGGTCCG
ATAACTGACCAAGTAACATGGTTATCACTaGATGTCCGCCAGACGTGTGCAAACCAACCCGGGAGTTACGTCACT
AATCCTTCGCTACGTCGTGAAGATATTTACTTGTGAATATCGAGGGTAATAAGATAATAGACTGTGACTAGTATTG
CCAGACTGTCGCTACCTGCAACACATAACTATCCTGAGGTTACTGCATAGTACTGATTACACCCGAGTCAAAATT
TCTAACTTCTAACATGTACCTAGTAACCAGCTCAATAATTATGTCAGAATATAGCTCTGGGAACCCTCGGACAATT
ATGATACACGGTATTAATATCTTGCTTGCGTTAGCCACTTCTCATCTTTGGATACCGATTCTATTTTGCATAGCAGT
TCCTTTTACACATATAAGAATTTCGCCATAGGTATGCTTAAGGAAGTCGAGATTGCGAACCATTACCGAGACTATG
GCTTCATGTGGTGATTTCACCCGACCCACCCTTGGCGCCAGCTTTACGCAGCTTCCTGACGATACGTGGTGTAA
CGTTGTGTTTGGCAATGGAAACCGAGATCAACTATTTCTAATGCTGATATAGCAGAGTCTCGCGTCTATCATACG
CAAGTCGCACGTCATTTTCGAGAGCAGCGTAAGACTCTGAAGGTCATGAGCCCAGATGTTATTACCCTCTACCTA
TAAACATCAAAATTGTAGTCGTTTTACAGTCCATCGTCGCTCCAGAGCGAAGATTAAGGTTAGATCTAGATTATCT
TTGCACGTGTGGACCGACGCAGCTGGGGCTCTAGCTCCACTACGGTTACGAAACTGCTGAACGATCTGGTCCA
CTTCAAGATTCACACATCGTTTCATTCTTTGGACAACCAACACTCTCAGTCAGAGTTTCGAGTATAATAATTCTTCC
GCGCTAGGGTAAAAAGCAGATATGGGGAGACATTCCGGGCTTTTGAGCCGATACACTAAGCACTTGACATACTC
ACATCAGTAGAGGTTAACATTCATGACTATCACGCGCTGCAG

Table S2. Staple oligonucleotide sequences for bare ICO NANP

Staple #	DNA sequence (5' - 3')
2	ACATCTGGATGGCGAAATTCTTATATGTGGTAGAGGGTAATA
3	GCTCATGACCAAGCATACCT
4	GTTCGCAATCTTTTTTCGACTTCCTTTTCAGAGTCTTTTTTTACGCTGCTCT
5	CGGTAATGTCACCACATGAAGCCGTCCGAGG
6	GTTCCCAGACCGTGTATCATAATTATAGTCT
7	GCGAGACTCTGTTTTTCTATATCAGCCCAAACACAACTTTTTGTTACACCACCCAAGGGTGGGTTTTTTCGGGTGAAA
8	AGCTGGCGGTATCGTCAGGAAGCTAACCTCA
10	GGATAGTTAATCAGTACTATGCAGTGCGTAA
11	TTCCATTGATTAGAAATAGTTGAGTAGCGAA
12	GGATTAGTTAAATATCTTCACGACTCTCGGT
13	TGATAGACCGAAAATGACGTGCGACACGTGC
14	AAAGATAAAGCTGCGTCGGTCCACTTGCGTA
15	CTACAATTTTGTTTTTATGTTTATAGTAAAAGGAACTTTTTTGCTATGCAAATTTTTATAGGTTTTTTTAATGTCATG
16	GTCAAGTGCGACGATGGACTGTAAAACGACTGATGTGAGTAT
17	CTTAGTGTATCGCTCTGGAG
18	TCTAGATCTAATTTTTCCTTAATCTTCGGCTCAAAAGTTTTTCCCGGAATGT
19	TTTCGTAAAGTGGACCAGATCGTACATCTAG
20	TGATAACCTGCACACGTCTGGCGGTCAGCAG
21	CAAAGAATCTGACTGAGAGTGTTTGATAACA
22	CTGCGGCCCTGCCATAACCATGAGGGTTGTC
23	AGCGCGGACTCCCCATATCTGCTTACCCGCC
24	GCGCTTAAGCGCGTAACCACCACTTTACCCT
25	CAACGTCAAAGTTTTTGGCGAAAAACAGTCATGAATGTTTTTTTAACCTCTA
26	GATGGCCCTGCGCTCCTGCAGCGCGTGATCGTCTATCAGGGC
27	ACTACGTGAATTAATTGCGT
28	TGCGCCGCTACTTTTTAGGGCGCACACCATCACCCTATTTTTATCAAGTTTT
29	GAACCCTAAAGTTTTTGGAGCCCCCGCGTGGCGAGAATTTTTAGGAAGGGAAGGCAAGTGTAGTTTTTCGGTCACGCT
30	AGGGCGCTGAAAGCGAAAGGAGCTAACCGCT
31	TTTTTGCAGGAGGACCGAAGGAGCGGGCGCT
32	CCGGCGAAATTTAGAGCTTGACGATCATATG
33	TACCCCGGTGAGCGTCAGACCCCAGGGAAAG
34	CTAAATCGTTGGGGTCGAGGTGCCAGACCGA
35	GATAGGGTATAAATCAAAAGAATGTAAAGCA
36	CTTAGACGACTATTAAAGAACGTGGACTCATAATAATGGTTT
37	TCAGGTGGCAACAAGAGTCC
38	TGAGTGTTGTTTTTTTCCAGTTTGGACTTTTCGGGGATTTTTAATGTGCGCG
39	ATTCAAATATGTTTTTTATCCGCTCACGTAGTTATCTTTTTTACACGACGGGCCGAAATCGGCTTTTTAAAATCCCTT
40	ATGGATGAGCTCATTTTTTAACCAATAGGGAGTCAGGCAACT
41	ACGAAATAGATGTTAAATCA
42	TAACGTGAGTTTTTTTTTCGTTCCACTTGTAATTCGCTTTTTGTTAAATTTTCAGATCGCTGATTTTTGATAGGTGCC
43	AAATCCCTCTTTTTGATAATCTCGAACCGGA
44	GCTGAATGTCGCCTTGATCGTTGGATGACCA
45	CAAGTTTACTCTTTTTATATATACTTAAAAGGATCTATTTTTGGTGAAGATC
46	TTTAATTTTAGATTGATTTAAAACTAGCTTC
47	CCGGCAACTGGCGAACTACTTACTCTTCATT
48	TGTCAGACTCACTGATTAAGCATGGAGCCGG
49	TGAGCGTGTATTGCTGATAAATCTTGGTAAC
50	CTGATAAAAGATGGTAAGCCCTCCCGTATTGAGACAATAACC
51	TGCTTCAATACACTGGGGCC
52	GGTCTCGCGGTTTTTTATCATTGCAGATATTGAAAAATTTTTGGAAGAGTATCCTTTTTTGCGTTTTTGCATTTTGCC

53	TGAAAGTAAAATTTTTGATGCTGAAGGGCCCTTCCGGTTTTTCTGGCTGGTT
54	ACGAGTGGTGCAGGACCACTTATGCGCTCATCAGTTGGGTGC
55	GTTACATCGACGGATAAAGT
56	AATTAATAGACTTTTTTGGATGGAGGACTGGATCTCATTTTTACAGCGGTAA
57	TTCCAATGATGTTTTTAGCACTTTTAATTGACGCCGGTTTTTGCAAGAGCAAAACGTTGCGCATTT TTAACTATTAAC
58	CAATGGCAACCTCGGTCGCC
59	GCCTGTAGGCATACACTATTCTCAGAATGGTGACACCACGAT
60	CAACATGGGGGTTTTTATCATGTAACAAGCCATACCATTTTTAACGACGAGCACTTGGTTGAGTTT TTTACTCACCAG
61	CAGTAAGAGAATTTTTTTATGCAGTGAACTTACTTCTTTTTTGACAACGATC
62	TGGCATGATCACAGAAAAGCATCTCATTGTG
63	CAATCCTAGACCACGTGTATTCATTTACGGA
64	TATCCCGTAAGTTCTGCTATGTGGGAAAGCA
65	TGGTAGCATCGTTAATGACCCAACGCGGTAT
66	AGAACGTTGATCCTTGAGAGTTTTCTTCAAA
67	CGGGTAGGAGCACCTGTATCGGCCGCCCCGA
68	AACGCTGGTTCCTGTTTTTGCTCGTGAAGAA
69	CCAGGGAAAAGTCAAATCCTATCTACCCAGA
70	CCTTATTCGAGTATTCAACATTTCTGGCTCA
71	ACAACAGAAATCTGGCGCCCGGGCGTGTCGC
72	CTAAATACGAACCCCTATTTGTTTAGCCTCT
73	CCACCCAATGTGACCTTAAACGAATATTTTT
74	CCAAAGATAAGGGCCTCGTGATACGCCTAATAGAATCGGTAT
75	GAGAAGTGGCAATTCGACGA
76	GAATAAGTAAATTTTTTTACATGCGAGAGTTTGAGGGTTTTTGACGACGACG
77	TAACGCAAGCATTTTTAGATATTAATAGCTATATTCTTTTTTGACATAATTAAAGAGACAAAGTTTTT CTTTAAACAA
78	TAGGTACACTGAATCTGTCTATACAAACTTTGAGCTGGTTAC
79	TGTTAGAAGTACCTTGCACT
80	AAGCCGCATAATTTTTGTGTTGTAGAAACCAGGTACGTTTTTAGCGAACGAA
81	TAGAAATTTTGTTTTTACTCGGGTGTATGTGTTGCAGTTTTTGTAGCGACAGATGTTCGCCCGTTT TTCATTTTGCGC
82	TTGATACTCTTCTGGCAATA
83	GCGTTGTTCTAGTCACAGTCTATTATCTTCCTAGATTAGAAG
84	ATGTTACTTGGTTTTTTCAGTTATCGCTTTAAAAGAATTTTTAAGAGGTTGGAATACGTATCTTTTTT TCCCTATATT
85	ATTACCCTCGATTTTTTATTCACAAGGACGTAACTCCTTTTTCGGGTTGGTT

Table S3. Modified staple sequences

AF647-modified staples

ICO particles for flow cytometry assays were labeled only with a single AF647 dye using staple 3. For confocal microscopy, ICO particles were modified with six AF647 dyes for higher detection sensitivity.

Staple #	DNA sequence (5' - 3')
3	/AF647-**TT**GCTCATGACCAAGCATACCT
27	/AF647-**TT**ACTACGTGAATTAATTGCGT
51	/AF647-**TT**TGCTTCAATACACTGGGGCC
58	/AF647-**TT**CAATGGCAACCTCGGTCGCC
79	/AF647-**TT**TGTTAGAAGTACCTTGCACT
82	/AF647-**TT**TTGATACTCTTCTGGCAATA

Staples modified with ligands

DNA sequence complementary to BC2 and modified with either cholesterol, TriGalNAc, Palmitate or C18 ligands. X in the sequence indicates ligand position.

Staple #	DNA sequence (5' - 3')
Rev. BC2	**X-**TCTTGTGGACGTGCATCCAT

Staples for ICO with 1 cholesterol (ICO-1C) or 1 TriGalNAc (ICO-1T)

Staple #	DNA sequence (5' - 3')
2-BC2	ACATCTGGATGGCGAAATTCTTATATGTGGTAGAGGGTAATACCCATGGATGCACGTCCACAAGA

Staples for ICO with 6 TriGalNAc (ICO-6T) or 6 lipids (cholesterol, C18 and palmitate) in dispersed configuration

For ICO of varying ligand copies following staples were used for hybridization with DNA-modified ligand; ICO-1x: staple 2; ICO-2x: staple 2 and 21; ICO-3x: staples 2, 21, and 35; ICO-4x: staples 2, 21, 35, and 47; ICO-5x: staples 2, 21, 35, 47 and 71; and ICO-6x:
Sequence for DNA extension BC2 is indicated in green color.

Staple #	DNA sequence (5' - 3')
2-BC2	ACATCTGGATGGCGAAATTCTTATATGTGGTAGAGGGTAATACCCATGGATGCACGTCCACAAGA
21-BC2	CAAAGAATCTGACTGAGAGTGTTTGATAACACCCATGGATGCACGTCCACAAGA
35-BC2	GATAGGGTATAAATCAAAAGAATGTAAAGCACCCATGGATGCACGTCCACAAGA
47-BC2	CCGGCAACTGGCGAACTACTTACTCTTCATTCCCATGGATGCACGTCCACAAGA
71-BC2	ACAACAGAAATCTGGCGCCCGGGCGTGTCGCCCCATGGATGCACGTCCACAAGA
83-BC2	GCGTTGTTCTAGTCACAGTCTATTATCTTCCTAGATTAGAAGCCCATGGATGCACGTCCACAAGA

Staples for ICO with up to 5 lipids clustered configuration

For ICO of varying lipid copies following staples were used for hybridization with DNA-modified lipids; ICO-1x: staple 2; ICO-2x: staple 2 and 15; ICO-3x: staples 2, 15, and 26; ICO-4x: staples 2, 15, 26 and 36; and ICO-5x: staples 2, 15, 26, 36 and 74. Sequence for DNA extension BC2 is indicated in green color.

Staple #	DNA sequence (5' - 3')
2-BC2	ACATCTGGATGGCGAAATTCTTATATGTGGTAGAGGGTAATACCCATGGATGCACGTCCACAAGA
15-BC2	GTCAAGTGCGACGATGGACTGTAAAACGACTGATGTGAGTATCCCATGGATGCACGTCCACAAGA
26-BC2	GATGGCCCTGCGCTCCTGCAGCGCGTGATCGTCTATCAGGGCCCCATGGATGCACGTCCACAAGA
36-BC2	CTTAGACGACTATTAAAGAACGTGGACTCATAATAATGGTTTCCCATGGATGCACGTCCACAAGA
74-BC2	CCAAAGATAAGGGCCTCGTGATACGCCTAATAGAATCGGTATCCCATGGATGCACGTCCACAAGA

Staples for ICO with 30 TriGalNAc (ICO-30T)

Staple #	DNA sequence (5' - 3')
2-BC2	ACATCTGGATGGCGAAATTCTTATATGTGGTAGAGGGTAATACCCATGGATGCACGTCCACAAGA
6-BC2	GTTCCCAGACCGTGTATCATAATTATAGTCTCCCATGGATGCACGTCCACAAGA
8-BC2	AGCTGGCGGTATCGTCAGGAAGCTAACCTCACCCATGGATGCACGTCCACAAGA
10-BC2	TTCCATTGATTAGAAATAGTTGAGTAGCGAACCCATGGATGCACGTCCACAAGA
13-BC2	AAAGATAAAGCTGCGTCGGTCCACTTGCGTACCCATGGATGCACGTCCACAAGA

15-BC2	GTCAAGTGCGACGATGGACTGTAAAACGACTGATGTGAGTATCCCATGGATGCACGTCCACAAGA
19-BC2	TTTCGTAAAGTGGACCAGATCGTACATCTAGCCCATGGATGCACGTCCACAAGA
21-BC2	CAAAGAATCTGACTGAGAGTGTTTGATAACACCCATGGATGCACGTCCACAAGA
23-BC2	AGCGCGGACTCCCCATATCTGCTTACCCGCCCCCATGGATGCACGTCCACAAGA
26-BC2	GATGGCCCTGCGCTCCTGCAGCGCGTGATCGTCTATCAGGGCCCCATGGATGCACGTCCACAAGA
31-BC2	TTTTTGCAGGAGGACCGAAGGAGCGGGCGCTCCCATGGATGCACGTCCACAAGA
33-BC2	TACCCCGGTGAGCGTCAGACCCCAGGGAAAGCCCATGGATGCACGTCCACAAGA
35-BC2	GATAGGGTATAAATCAAAAGAATGTAAAGCACCCATGGATGCACGTCCACAAGA
36-BC2	CTTAGACGACTATTAAAGAACGTGGACTCATAATAATGGTTTCCCATGGATGCACGTCCACAAGA
40-BC2	ATGGATGAGCTCATTTTTTAACCAATAGGGAGTCAGGCAACTCCCATGGATGCACGTCCACAAGA
44-BC2	GCTGAATGTCGCCTTGATCGTTGGATGACCACCCATGGATGCACGTCCACAAGA
47-BC2	CCGGCAACTGGCGAACTACTTACTCTTCATTCCCATGGATGCACGTCCACAAGA
49-BC2	TGAGCGTGTATTGCTGATAAATCTTGGTAACCCCATGGATGCACGTCCACAAGA
50-BC2	CTGATAAAAGATGGTAAGCCCTCCCGTATTGAGACAATAACCCCCATGGATGCACGTCCACAAGA
54-BC2	ACGAGTGGTGCAGGACCACTTATGCGCTCATCAGTTGGGTGCCCCATGGATGCACGTCCACAAGA
59-BC2	GCCTGTAGGCATACACTATTCTCAGAATGGTGACACCACGATCCCATGGATGCACGTCCACAAGA
63-BC2	CAATCCTAGACCACGTGTATTCATTTACGGACCCATGGATGCACGTCCACAAGA
65-BC2	TGGTAGCATCGTTAATGACCCAACGCGGTATCCCATGGATGCACGTCCACAAGA
67-BC2	CGGGTAGGAGCACCTGTATCGGCCGCCCCGACCCATGGATGCACGTCCACAAGA
69-BC2	CCAGGGAAAAGTCAAATCCTATCTACCCAGACCCATGGATGCACGTCCACAAGA
71-BC2	ACAACAGAAATCTGGCGCCCGGGCGTGTCGCCCCATGGATGCACGTCCACAAGA
73-BC2	CCACCCAATGTGACCTTAAACGAATATTTTTCCCATGGATGCACGTCCACAAGA
74-BC2	CCAAAGATAAGGGCCTCGTGATACGCCTAATAGAATCGGTATCCCATGGATGCACGTCCACAAGA
78-BC2	TAGGTACACTGAATCTGTCTATACAAACTTTGAGCTGGTTACCCCATGGATGCACGTCCACAAGA
83-BC2	GCGTTGTTCTAGTCACAGTCTATTATCTTCCTAGATTAGAAGCCCATGGATGCACGTCCACAAGA

Staples for ICO-6C with 2 siRNAs

Sequence for DNA extension BC1 is indicated in purple color.

Staple #	DNA sequence (5' - 3')
10-BC1	TTCCATTGATTAGAAATAGTTGAGTAGCGAACCCGATACTGCCATAGACGGCTG
36-BC1	CTTAGACGACTATTAAAGAACGTGGACTCATAATAATGGTTTCCCGATACTGCCATAGACGGCTG

Staples for ICO-6C with 6 siRNAs

Sequence for DNA extension BC1 is indicated in purple color.

Staple #	DNA sequence (5' - 3')
10-BC1	TTCCATTGATTAGAAATAGTTGAGTAGCGAACCCGATACTGCCATAGACGGCTG
23-BC1	AGCGCGGACTCCCCATATCTGCTTACCCGCCCCCGATACTGCCATAGACGGCTG
36-BC1	CTTAGACGACTATTAAAGAACGTGGACTCATAATAATGGTTTCCCGATACTGCCATAGACGGCTG
50-BC1	CTGATAAAAGATGGTAAGCCCTCCCGTATTGAGACAATAACCCCCGATACTGCCATAGACGGCTG
65-BC1	TGGTAGCATCGTTAATGACCCAACGCGGTATCCCGATACTGCCATAGACGGCTG
74-BC1	CCAAAGATAAGGGCCTCGTGATACGCCTAATAGAATCGGTATCCCGATACTGCCATAGACGGCTG

Staples for ICO-6C with 12 siRNAs

Sequence for DNA extension BC1 is indicated in purple color.

Staple #	DNA sequence (5' - 3')
6-BC1	GTTCCCAGACCGTGTATCATAATTATAGTCTCCCGATACTGCCATAGACGGCTG
10-BC1	TTCCATTGATTAGAAATAGTTGAGTAGCGAACCCGATACTGCCATAGACGGCTG

15-BC1	GTCAAGTGCGACGATGGACTGTAAAACGACTGATGTGAGTATCCCGATACTGCCATAGACGGCTG
23-BC1	AGCGCGGACTCCCCATATCTGCTTACCCGCCCCCGATACTGCCATAGACGGCTG
31-BC1	TTTTTGCAGGAGGACCGAAGGAGCGGGCGCTCCCGATACTGCCATAGACGGCTG
36-BC1	CTTAGACGACTATTAAAGAACGTGGACTCATAATAATGGTTTCCCGATACTGCCATAGACGGCTG
44-BC1	GCTGAATGTCGCCTTGATCGTTGGATGACCACCCGATACTGCCATAGACGGCTG
50-BC1	CTGATAAAAGATGGTAAGCCCTCCCGTATTGAGACAATAACCCCCGATACTGCCATAGACGGCTG
59-BC1	GCCTGTAGGCATACACTATTCTCAGAATGGTGACACCACGATCCCGATACTGCCATAGACGGCTG
65-BC1	TGGTAGCATCGTTAATGACCCAACGCGGTATCCCGATACTGCCATAGACGGCTG
69-BC1	CCAGGGAAAAGTCAAATCCTATCTACCCAGACCCGATACTGCCATAGACGGCTG
74-BC1	CCAAAGATAAGGGCCTCGTGATACGCCTAATAGAATCGGTATCCCGATACTGCCATAGACGGCTG

Staples for ICO or ICO-6C with 24 siRNAs

Sequence for DNA extension BC1 is indicated in purple color.

Staple #	DNA sequence (5' - 3')
6-BC1	GTTCCCAGACCGTGTATCATAATTATAGTCTCCCGATACTGCCATAGACGGCTG
8-BC1	AGCTGGCGGTATCGTCAGGAAGCTAACCTCACCCGATACTGCCATAGACGGCTG
10-BC1	TTCCATTGATTAGAAATAGTTGAGTAGCGAACCCGATACTGCCATAGACGGCTG
13-BC1	AAAGATAAAGCTGCGTCGGTCCACTTGCGTACCCGATACTGCCATAGACGGCTG
15-BC1	GTCAAGTGCGACGATGGACTGTAAAACGACTGATGTGAGTATCCCGATACTGCCATAGACGGCTG
19-BC1	TTTCGTAAAGTGGACCAGATCGTACATCTAGCCCGATACTGCCATAGACGGCTG
23-BC1	AGCGCGGACTCCCCATATCTGCTTACCCGCCCCCGATACTGCCATAGACGGCTG
26-BC1	GATGGCCCTGCGCTCCTGCAGCGCGTGATCGTCTATCAGGGCCCCGATACTGCCATAGACGGCTG
31-BC1	TTTTTGCAGGAGGACCGAAGGAGCGGGCGCTCCCGATACTGCCATAGACGGCTG
33-BC1	TACCCCGGTGAGCGTCAGACCCCAGGGAAAGCCCGATACTGCCATAGACGGCTG
36-BC1	CTTAGACGACTATTAAAGAACGTGGACTCATAATAATGGTTTCCCGATACTGCCATAGACGGCTG
40-BC1	ATGGATGAGCTCATTTTTTAACCAATAGGGAGTCAGGCAACTCCCGATACTGCCATAGACGGCTG
44-BC1	GCTGAATGTCGCCTTGATCGTTGGATGACCACCCGATACTGCCATAGACGGCTG
49-BC1	TGAGCGTGTATTGCTGATAAATCTTGGTAACCCCGATACTGCCATAGACGGCTG
50-BC1	CTGATAAAAGATGGTAAGCCCTCCCGTATTGAGACAATAACCCCCGATACTGCCATAGACGGCTG
54-BC1	ACGAGTGGTGCAGGACCACTTATGCGCTCATCAGTTGGGTGCCCCGATACTGCCATAGACGGCTG
59-BC1	GCCTGTAGGCATACACTATTCTCAGAATGGTGACACCACGATCCCGATACTGCCATAGACGGCTG
63-BC1	CAATCCTAGACCACGTGTATTCATTTACGGACCCGATACTGCCATAGACGGCTG
65-BC1	TGGTAGCATCGTTAATGACCCAACGCGGTATCCCGATACTGCCATAGACGGCTG
67-BC1	CGGGTAGGAGCACCTGTATCGGCCGCCCCGACCCGATACTGCCATAGACGGCTG
69-BC1	CCAGGGAAAAGTCAAATCCTATCTACCCAGACCCGATACTGCCATAGACGGCTG
73-BC1	CCACCCAATGTGACCTTAAACGAATATTTTTCCCGATACTGCCATAGACGGCTG
74-BC1	CCAAAGATAAGGGCCTCGTGATACGCCTAATAGAATCGGTATCCCGATACTGCCATAGACGGCTG
78-BC1	TAGGTACACTGAATCTGTCTATACAAACTTTGAGCTGGTTACCCCGATACTGCCATAGACGGCTG

Table S4. siRNA sequences used in this study

Antisense strand (AS) and sense strand (SS) sequences for siRNA or TriGalNAc-siRNA (TGAL-siRNA) against human and mouse *ALDH2* gene were provided by Novo Nordisk.[3] To facilitate formulation into NANPs, the SS sequence was extended with a 20nt long modified RNA strand complementary to BC1.

MeMOPu indicates 5' 4'-*O*-methylphosphonate modified uridine. Capital letters indicate DNA nucleotides, “m” and “f” indicates 2′-O-methyl (2′-OMe) and 2′-deoxy-2′-fluoro (2′-F) ribosesugar modifications, respectively. “*" indicates phosphorothioate (PS) linkage, and ademA-GalNAc refers to GalNAc-modified adenine phosphoramidites, whereas ademG is NH_2 modified guanine phosphoramidites which was used to facilitate fluorophore labelling. ALDH2: Aldehyde dehydrogenase 2.

**Compound**	**Strand**	**Sequence (5' - 3')**
siRNA	AS	MeMOPu*fA*fAfAfCmUfGmAmGfUmUmUmCfAmUfCmCmAfCmC*mG*mG
	SS	mG*mGfUmGfGmAmUfGmAfAmAfCfUmCfAmGfUmUmUmAmGmCmAmGmCmCmG<br>mAmAmAmGmGmCmUmGmC
TriGalNAc-<br>siRNA	AS	MeMOPu*fA*fAfAfCmUfGmAmGfUmUmUmCfAmUfCmCmAfCmC*mG*mG
	SS	mG*mGfUmGfGmAmUfGmAfAmAfCfUmCfAmGfUmUmUmAmGmCmAmGmCmCmG<br>[ademA-GalNAc][ademA-GalNAc][ademA-GalNAc]mGmGmCmUmGmC
siRNA-<br>rev. BC1	AS	MeMOPu*fA*fAfAfCmUfGmAmGfUmUmUmCfAmUfCmCmAfCmC*mG*mG
	SS	mG*mGfUmGfGmAmUfGmAfAmAfCfUmCfAmGfUmUmUmAmCmAmGmCmCmGmU<br>mCmUmAmUmGmGmCmAmGmUmAmUmC
5'-NH_2<br>siRNA-rev.<br>BC1	AS	MeMOPu*fA*fAfAfCmUfGmAmGfUmUmUmCfAmUfCmCmAfCmC*mG*mG
	NH_2 -SS-<br>BC1	[ademG]*mGfUmGfGmAmUfGmAfAmAfCfUmCfAmGfUmUmUmAmCmAmGmCmCm<br>GmUmCmUmAmUmGmGmCmAmGmUmAmUmC
5'-NH_2<br>TriGalNAc-<br>siRNA	AS	MeMOPu*fA*fAfAfCmUfGmAmGfUmUmUmCfAmUfCmCmAfCmC*mG*mG
	SS	[ademG]*mGfUmGfGmAmUfGmAfAmAfCfUmCfAmGfUmUmUmAmGmCmAmGmCm<br>CmG[ademA-GalNAc][ademA-GalNAc][ademA-GalNAc]mGmGmCmUmGmC

Table S5. Tetrahedron DNA sequences used in this study

Tetrahedron DNA strands[1] were extended with DNA sequence BC1 (strand number-BC1) for hybridization of ligand-modified DNA strands complementary to BC1. Strand 6 was purchased with a 5' AF647 dye for detection purposes. BC1 sequence is indicated in green.

**Strand #**	**DNA sequence (5' - 3')**
1	GTCTGAGGCAGTTGAGAGATCTCGAACATTCC
2	TAAGTCTGAAGATCCATTTATCACCAGCTGCTGCACGCCATAGTAGACGTATCACCTGTCC
3	AGCTACTGCTACACGAGGATCTCAGACTTAGGAATGTTCGAGATCACATGCGAGGACTCGGTCA<br>ATACCGTACTAACGATACAGATCAA
4	AGCTACTGCTACACGAGGATCTCAGACTTAGGAATGTTCGAGATCACATGCGAGGACTCGGTCAT<br>ACCGTACTAACGATACAGATCAA
5	ATGCCCATC CGG CTC ACT ACT ATG GCG TGC AG
6	CGAGTCCTCGCATGACTCAACTGCCTCAGACGGACAGGTGATACGAGAGCCGGATGGGCATGC<br>TCTTCCCGTAGAGACGGTATTGGACATGAT
1-BC1	ATGCCCATC CGG CTC ACT ACT ATG GCG TGC AG
2-BC1	TAAGTCTGAAGATCCATTTATCACCAGCTGCTGCACGCCATAGTAGACGTATCACCTGTCCCC<br>CGATACTGCAGACGGCTG
3-BC1	AGCTACTGCTACACGAGGATCTCAGACTTAGGAATGTTCGAGATCACATGCGAGGACTCGGTCA<br>ATACCGTACTAACGATACAGATCAACCCGATACTGCCATAGACGGCTG
4-BC1	CAGCTGGTGATAAAACGTGTAAGTAGCTTTGATCTGTAATCGACTCTACGGGAAGAGCCCCGATA<br>CTGCCATAGACGGCTG
6-BC1	CGAGTCCTCGCATGACTCAACTGCCTCAGACGGACAGGTGATACGAGAGCCGGATGG<br>GCATGCTCTTCCCGTAGAGACGGTATTGGACATGATCCCGATACTGCCATAGACGGCTG
5-AF647	AF647- ATGCCCATCCGGCTCACTACTATGGCGTGCAG

Table S6. Holliday junction and DNA duplex sequences.

Strand #	DNA sequence (5' - 3')
Duplex-1	GATACTGCCATAGACGGCTG
Duplex-2	CAGCCGTCTATGGCAGTATC
Duplex-1-DBCO	/5DBCON/GATACTGCCATAGACGGCTG
Duplex-2-DBCO	/5DBCON/CAGCCGTCTATGGCAGTATC

Strand #	DNA sequence (5' - 3')
HJ-Q1	C+CG+TCCT+GA+GCC
HJ-Q2	CA+CA+GTG+GA+CGG
HJ-Q3	G+GC+TCACC+GA+TC
HJ-Q4	GA+TC+GGAC+TG+TG
HJ-Q1-DBCO	/5DBCON/C+CG+TCCT+GA+GCC
HJ-Q2-DBCO	/5DBCON/CA+CA+GTG+GA+CGG
HJ-Q3-DBCO	/5DBCON/G+GC+TCACC+GA+TC

Supporting References

(1) Lee, H.; Lytton-Jean, A. K.; Chen, Y.; Love, K. T.; Park, A. I.; Karagiannis, E. D.; Sehgal, A.; Querbes, W.; Zurenko, C. S.; Jayaraman, M.; et al. Molecularly self-assembled nucleic acid nanoparticles for targeted in vivo siRNA delivery. *Nat Nanotechnol* **2012**, *7* (6), 389-393. DOI: 10.1038/nnano.2012.73.

(2) Andersen, V. L.; Vinther, M.; Kumar, R.; Ries, A.; Wengel, J.; Nielsen, J. S.; Kjems, J. A self-assembled, modular nucleic acid-based nanoscaffold for multivalent theranostic medicine. *Theranostics* **2019**, *9* (9), 2662-2677. DOI: 10.7150/thno.32060.

(3) Ganesh, S.; Kim, M. J.; Lee, J.; Feng, X.; Ule, K.; Mahan, A.; Krishnan, H. S.; Wang, Z.; Anzahaee, M. Y.; Singhal, G.; et al. RNAi mediated silencing of STAT3/PD-L1 in tumor-associated immune cells induces robust anti-tumor effects in immunotherapy resistant tumors. *Mol Ther* **2024**, *32* (6), 1895-1916. DOI: 10.1016/j.ymthe.2024.03.035.
